# Intestinal Fucosylation: A Key Regulatory Hub in Homeostasis and Disease Pathogenesis

**DOI:** 10.3390/biom16091226

**Published:** 2026-08-24

**Authors:** Zhishan Xu, Dingbo Song, Fangqi Hu, Qiuhan Liang, Mengyao Zhang, Chao Lei, Jinyuan Li, Haiyi Guo, Zhongbin Deng, Zishan Yang

**Affiliations:** 1 Department of Immunology, School of Basic Medical Sciences, Henan Medical University, Xinxiang 453003, China; 201070@hamu.edu.cn (Z.X.); 20231101223@stu.hamu.edu.cn (D.S.); 20241100109@stu.hamu.edu.cn (F.H.); 20221390128@stu.hamu.edu.cn (Q.L.); 20241103102@stu.hamu.edu.cn (M.Z.); 20231100906@stu.hamu.edu.cn (J.L.); 20241103415@stu.hamu.edu.cn (H.G.); 2 Xinxiang Key Laboratory of Tumor Vaccine and Immunotherapy, School of Basic Medical Sciences, Henan Medical University, Xinxiang 453003, China; 3 Department of Surgery, Division of Immunotherapy, University of Louisville, Louisville, KY 40202, USA; chao.lei@louisville.edu; 4 Brown Cancer Center, University of Louisville, Louisville, KY 40202, USA

**Keywords:** fucosylation, gut, mucosal protection, inflammatory bowel disease, colorectal cancer

## Abstract

Inflammatory bowel disease (IBD) and colorectal cancer (CRC) are heterogeneous intestinal disorders that pose significant threats to human health and share common pathological features, including intestinal mucosal barrier disruption and gut microbiota dysbiosis, in which fucosylation acts as a critical regulatory mediator. Fucosylation is a highly conserved post-translational glycosylation modification involving the enzymatic transfer of fucose residues to glycoproteins and glycolipids. This tightly regulated process plays essential roles in maintaining intestinal homeostasis, mediating host–microbiota interactions and regulating immune responses. This review adopts a physiology-to-pathology framework, delineating fucosylation’s operational principles in healthy intestines and its dysregulation in IBD and CRC. It summarizes the spatial distribution of fucosylation, its regulatory mechanisms, and its roles in disease pathogenesis, and also discusses its potential as a diagnostic biomarker and therapeutic target. Finally, this review highlights future research directions to bridge mechanistic insights with clinical translation, emphasizing the promise of fucosylation in the precision diagnosis and treatment of intestinal disorders.

## 1. Introduction

Inflammatory bowel disease (IBD) and colorectal cancer (CRC) are heterogeneous intestinal disorders that represent increasing global health challenges, particularly in the context of rapidly changing lifestyles and environmental exposures. IBD, which primarily comprises Crohn’s disease (CD) and ulcerative colitis (UC), represents a group of idiopathic, chronic, relapsing inflammatory disorders that affect approximately 0.3–0.5% of the global population. The increasing incidence and prevalence of IBD have substantially contributed to the growing global healthcare burden associated with this disease [1,2]. CRC, a major malignancy of the digestive system accounting for 10% of all cancer diagnoses [3], is the gastrointestinal cancer with the highest lifetime risk [4]. In 2022, CRC ranked third in global cancer incidence and second in cancer-related mortality, with over 1.9 million new cases and 904,000 deaths worldwide [5,6]. Despite their distinct etiological backgrounds, IBD and CRC share several pathological features, including disruption of the intestinal mucosal barrier, gut microbiota dysbiosis, and impaired host–microbiota communication. These pathological processes collectively highlight fucosylation, a conserved post-translational modification, as a key regulatory mediator at the intestinal interface.

Fucosylation, defined as the enzymatic transfer of terminal fucose residues to glycoproteins and glycolipids by fucosyltransferases (FUTs), plays essential roles in cell–cell communication, immune recognition, signal transduction, and host–microbiota interactions [7]. The biosynthesis of fucose proceeds through two tightly regulated pathways: de novo synthesis and the salvage pathway. Products from both pathways converge to generate guanosine diphosphate-fucose (GDP-fucose), the universal donor for fucosylation reactions [8]. In the intestine, fucosylation constitutes a critical component of the multi-layered mucosal barrier, enhancing the structural stability and lubricity of secreted mucins, providing ligands for immune receptors, and constructing ecological niches for commensal microbiota [9,10]. However, a fundamental paradox remains unresolved: the same glycosylation modification can exert either protective or pathogenic effects depending on glycosidic linkage type, substrate identity, cellular context, and local microenvironment. Although numerous gene-centric studies have provided valuable insights, a comprehensive framework integrating these findings and explaining the context-dependent functions of intestinal fucosylation remains lacking.

Here, we advance the thesis that intestinal fucosylation constitutes a dynamic, spatially organized, and context-dependent regulatory—a molecular interface code that orchestrates host–microbiota mutualism, immune homeostasis, and barrier integrity under physiological conditions, yet becomes fundamentally dysregulated in IBD and CRC. To address this hypothesis, we employ a physiology-to-pathology framework, first describing the regulatory principles governing fucosylation in healthy intestinal biology and subsequently examining how these mechanisms are disrupted during disease development. Throughout this review, we distinguish correlative associations from causal mechanistic evidence, discuss unresolved controversies, and identify specific, testable knowledge gaps that currently hinder clinical translation.

## 2. Overview of Fucosylation

Fucosylation is a form of glycosylation in which guanosine GDP-fucose is attached to terminal glycans of glycoproteins and glycolipids via a tightly regulated enzymatic process involving various enzymes. GDP-fucose is a nucleotide sugar donor containing a six-carbon fucose moiety and serves as the primary substrate for fucosylation reactions occurring on cell surface glycoconjugates. According to the glycosidic linkage formed, fucosylation is classified into four major types: α1–2-, α1–3-, α1–4-, and α1–6-linked fucosylation.

### 2.1. The Biosynthesis of Fucose and Its Attachment to Glycoproteins and Glycolipids

**Figure 1:**
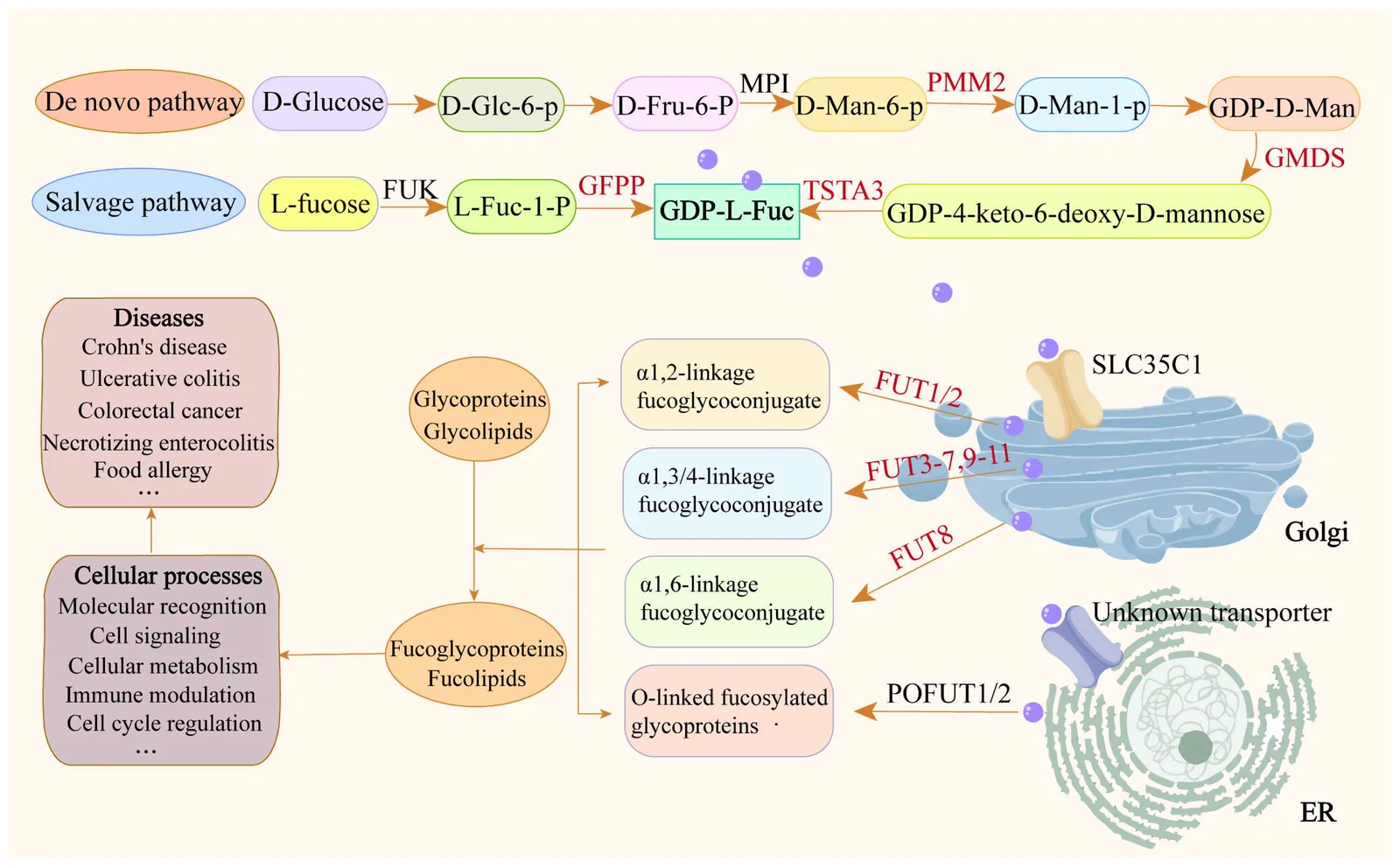
Overview of fucosylation. This figure illustrates the de novo and salvage pathways for GDP-fucose synthesis, its transport to the Golgi apparatus and the endoplasmic reticulum (ER), and downstream glycan modifications mediated by fucosyltransferases. The metabolites shown in the pathways and their chemical structures are detailed in the figure. SLC35C1 is a GDP-fucose transporter localized to the Golgi apparatus. The unknown transporter in the ER membrane is an uncharacterized factor responsible for ER O-fucosylation. Downstream fucosyltransferases (FUTs and POFUTs) catalyze distinct types of glycosidic linkages, including α1,2-, α1,3/4-, α1,6-, and O-linked fucosylation. Fucosylation plays critical roles in molecular recognition, cell signaling, metabolism, and immune modulation, and its dysregulation is implicated in inflammatory bowel disease, colorectal cancer, necrotizing enterocolitis, food allergy, and other pathologies.

Fucose is a six-carbon deoxy monosaccharide characterized by the absence of a hydroxyl group at the C-6 position. Although fucose exists in both L- and D-configurations, L-fucose is the predominant and biologically relevant form in living organisms [7]. L-fucose is known to be present in various microorganisms, as well as plant and animal tissues, serving as a major component of capsular polysaccharides in certain bacteria and polysaccharides in some marine algae. In humans, L-fucose is an important component of blood group antigens and is present in various oligosaccharides, including those found in human milk [8]. Under physiological conditions, fucose biosynthesis occurs primarily in the cytoplasm, whereas subsequent fucosylation reactions mainly take place in the Golgi apparatus through the coordinated action of multiple enzymes. This process is accomplished through two pathways: the de novo synthesis pathway (accounting for approximately 90%) and the salvage pathway [9]. The de novo pathway is initiated with D-mannose, which undergoes multiple reactions to form GDP-D-mannose. GDP-D-mannose is then converted to GDP-4-keto-6-deoxy-D-mannose by GDP-mannose 4,6-dehydratase, and finally transformed into GDP-L-fucose by the GDP-4-keto-6-deoxymannose 3,5-epimerase-4-reductase (FX, also known as TSTA3), a process that consumes NADPH and generates NADP^+^ [10]. The salvage pathway utilizes free L-fucose derived from extracellular sources or lysosomes. This fucose is phosphorylated by fucokinase to form L-fucose-1-phosphate, which is then combined with GTP by GDP-fucose pyrophosphorylase (GFPP) to generate GDP-L-fucose. This pathway is subject to feedback inhibition by GDP-L-fucose [9,11]. (Figure 1, Table 1).

GDP-fucose synthesized in the cytoplasm is transported into the Golgi lumen via the GDP-fucose transporter (GDP-L-Fuc Tr) located on the Golgi membrane. This transport process is coupled to the countertransport of GMP, an antiport mechanism that exchanges imported GDP-fucose for luminal GMP, the byproduct of fucosylation, since preloading of proteoliposomes with GMP stimulated GDP-fucose transport activity by 2- to 3-fold [12]. Defects in this transporter, as seen in leukocyte adhesion deficiency type II (LAD II) patients, lead to abnormal fucosylation. The gene Solute Carrier Family 35 Member C1 (SLC35C1) encodes the GDP-fucose transporter, providing the substrate for cell surface fucosylation. This transporter represents a critical step in fucose biosynthesis, ensuring the normal generation of fucosylated glycoproteins and glycolipids on the cell surface [13]. However, the transporter delivering GDP-fucose to the endoplasmic reticulum (ER) for POFUT1/2-catalyzed O-fucosylation remains unidentified in mammals [14].

Fucosylation, the attachment of fucose to glycoproteins and glycolipids, occurs primarily in the Golgi apparatus (with certain specific O-fucosylation events occurring in the rough endoplasmic reticulum). This process is catalyzed by FUTs, which exhibit catalytic specificity. These enzymes transfer fucose from the donor GDP-fucose to the terminal ends of glycan chains on glycoproteins or glycolipids via distinct glycosidic linkages, such as α1–2, α1–3, α1–4, or α1–6, thereby generating diverse fucosylated structures [11,15]. Fucosylation products with different linkage types exhibit differences in their physiological functions, participating in processes such as blood group antigen synthesis, immune cell adhesion, and tumor cell metastasis [15].

The structural diversity of fucosylated glycans is not only manifested in their glycan sequences and linkage types, but also in the types of glycan carriers (glycoproteins or glycolipids) to which they are attached. Importantly, these carrier types differentially influence the biological outcomes of pathogenic infection. For example, cholera toxin (CT) recognizes fucosylated glycans through its noncanonical binding pocket, and in intestinal epithelial cells, its engagement with fucosylated glycans on distinct carriers elicits completely opposing outcomes. Knockout of β-1,3-N-acetylglucosaminyltransferase 5 (B3GNT5) reduces specific fucosylated glycosphingolipids (GSLs), resulting in decreased CT binding but increased cellular sensitivity, indicating that these GSLs serve as decoy receptors. Conversely, knockout of β-1,3-galactosyltransferase 5 (B3GALT5) elevates the production of fucosylated glycoproteins, which simultaneously enhances both CT binding and cellular intoxication, identifying these glycoproteins as functional receptors for CT [13].

Therefore, the biosynthesis of fucose and its incorporation into glycoproteins and glycolipids is a strictly regulated process. This process is crucial for a wide range of cellular functions, including cell–cell communication, immune recognition, host–pathogen interactions, and tumorigenesis and progression.

### 2.2. Key Enzymes Involved in Fucosylation

Fucosylation involves the addition of fucose residues to glycoproteins and glycolipids, requiring the participation of numerous enzymes. The following section provides an overview of the major enzymes involved (Figure 1, Table 2).

#### 2.2.1. Phosphomannomutase 2 (PMM2)

PMM2, phosphomannomutase 2, is crucial for the biosynthesis of various glycoconjugates. As a member of the phosphoglucomutase superfamily, PMM2 is ubiquitously expressed across human tissues, with particularly high expression in the gastrointestinal tract [16]. PMM2 is localized in the cytoplasm, where it reversibly catalyzes the conversion of mannose-6-phosphate to mannose-1-phosphate, thereby supplying essential intermediates for glycosylation pathways. This reaction represents an essential upstream step in the de novo biosynthetic pathway leading to GDP-fucose production. Structurally, PMM2 typically functions as a homodimer, with each subunit undergoing conformational changes mediated by the relative movement of its core domain (catalytic) and cap domain (dimerization/activator binding), facilitating catalysis [17]. PMM2 activity is regulated at both translational and post-translational levels. Furthermore, PMM2 activity depends on its specific homodimeric structure and requires metal ions Mg^2+^ (including catalytic Mg-1 and structural Mg-2). Mg-1 functions as the catalytic core that directly participates in the phosphoryl transfer reaction, whereas Mg-2 serves as a structural rivet that maintains the conformational integrity of the active site, both of them are Mg^2+^) and activator sugar diphosphates (mannose or glucose), such as Glc-1,6-P_2_ [18,19]. Mutations in PMM2 represent the most common glycosylation disorder and are associated with defects in diverse glycosylation modifications, cell adhesion, and mitochondrial function. Disease-associated PMM2 mutations, including Cys9Tyr and Asp65Tyr substitutions, impair enzymatic activity by destabilizing protein structure and disrupting critical molecular interactions. Consequently, PMM2 deficiency represents the most common congenital disorder of glycosylation and is associated with impaired glycosylation, abnormal cell adhesion, mitochondrial dysfunction, and diverse clinical manifestations [18,19,20,21].

#### 2.2.2. GDP-Mannose Pyrophosphorylase (GDP-MP)

GDP-MP is a key enzyme in sugar metabolism. In humans, GDP-MP exists as a heterocomplex of GMPPA and GMPPB subunits, typically comprising eight GMPPA subunits and four GMPPB subunits. The enzyme’s activity is regulated by the interaction between GMPPA and GMPPB and is closely linked to cytosolic GDP-mannose levels. The GMPPA–GMPPB complexes catalyze the reaction of GTP and mannose-1-phosphate to form the GDP-mannose, which is an essential precursor for the de novo synthesis of GDP-fucose [22]. Functionally, GMPPB serves as the catalytic subunit, while GMPPA primarily acts as a regulatory subunit. The GMPPB-catalyzed GDP-mannose serves as a mannose donor for glycosylation or participates in generating various glycoconjugates (e.g., GDP-fucose, rhamnose), playing vital roles in cellular regulation. Loss of GMPPB function is strongly associated with muscular dystrophy and variable neurological symptoms [23,24].

#### 2.2.3. GDP-D-Mannose 4,6-Dehydratase (GMDS)

GMDS is one of the most important rate-limiting enzymes in the de novo GDP-L-fucose biosynthetic pathways [25]. The substrate GDP-D-mannose is a key intermediate for GDP-L-fucose production. GMDS catalyzes the conversion of GDP-D-mannose to GDP-4-keto-6-deoxymannose and plays a critical role in this catalytic process [26]. Deficiency in GMDS results in fucosylation defects and contributes to tumor immune evasion, such as resistance to the tumor necrosis factor-related apoptosis-inducing ligand (TRAIL) signaling pathway. Recent research also identifies GMDS as a novel binding partner for tankyrase 1. By inhibiting Poly (ADP-ribose) polymerase (PARP, an enzyme that uses NAD^+^ to transfer ADP-ribose onto target proteins) enzyme activity, GMDS helps maintain tankyrase 1 stability, potentially influencing cancer-associated pathways like telomere maintenance and Wnt signaling, highlighting its potential value as a cancer therapeutic target [27]. Previous studies have demonstrated that patients with LAD II display a marked reduction in GMD catalytic activity, which in turn compromises GDP-L-fucose biosynthesis. Of note, this enzymatic defect is not attributable to structural or genetic alterations within the GMD locus itself, but appears to originate from a dysfunctional upstream regulatory axis. Thus, whereas GMD inactivation is a key event in the metabolic cascade of LAD II, it should not be regarded as the genetic root cause of the disease [28,29].

#### 2.2.4. GDP-4-Keto-6-Deoxymannose-3,5,Epimerase-4-Reductase/FX (TSTA3/FX)

TSTA3, also known as the GDP-L-fucose synthase, was first isolated from human erythrocytes in 1976 [22]. It is a crucial enzyme synthesizing GDP-L-fucose using GDP-4-keto-6-deoxy-D-mannose as the substrate. FX is a bifunctional enzyme catalyzing two sequential reactions: first, an epimerase reaction altering carbons 3 and 5 of the hexose moiety of GDP-4-keto-6-deoxymannose, followed by an NADPH-dependent reductase reaction that reduces the 4-keto group [30]. The resultant GDP-L-fucose serves as the universal fucosyl donor for cellular fucosylation processes, which are critically involved in regulating multiple biological events including growth factor signaling transduction and cell–cell adhesion [29]. Structural and biochemical analyses have demonstrated that human FX binds NADPH via seven conserved amino acid residues, and the identification of its core catalytic residues provides promising molecular targets for the development of therapeutic strategies against inflammatory disorders, autoimmune diseases, and potentially certain malignancies. Inhibition of FX activity blocks GDP-L-Fuc synthesis, therefore suppressing fucosylation-dependent pathological processes like tumor invasion and inflammatory cell recruitment [27]. FX deficiency causes a complete interruption in GDP-L-fucose synthesis, which in turn suppresses Notch1./2 activation and downstream target gene Hes1 expression, impairs intestinal barrier function, disrupts gut microbiota structure, and enhances susceptibility to colitis, serrated lesions, adenocarcinomas, and right-sided colon cancer [29,31].

#### 2.2.5. Fucosyltransferase Family (FUT1–FUT11 and POFUT1/2)

Fucosyltransferases catalyze the transfer of fucose residues from GDP-fucose to specific glycoprotein or glycolipid acceptors, generating fucosylated glycoconjugates through the formation of defined glycosidic linkages and regulating diverse biological processes. The human genome encodes 13 FUTs (FUT1–11 and POFUT1,2), each exhibiting distinct tissue distribution and catalytic specificities. FUT1 and FUT2, localized to the Golgi apparatus, catalyze α1,2-fucosylation, involved in synthesizing ABH and Lewis blood group antigens. FUT3-FUT7 and FUT9-FUT11, localized to the Golgi apparatus, catalyze α1,3/α1,4-fucosylation to synthesize Lewis antigens (e.g., Le^a^, Le^b^, Le^x^) and their derivatives. FUT8, localized to the Golgi apparatus, is an α1,6-fucosyltransferase that catalyzes core fucosylation by attaching fucose via an α1,6-linkage to the innermost N-acetylglucosamine of N-glycans (occurring exclusively on N-glycans of glycoproteins) [9]. POFUT1 and POFUT2, located in the endoplasmic reticulum, catalyze O-fucosylation of serine/threonine residues within EGF-like domains [11,15]. Through the enzymatic activities, FUTs regulate the formation of fucosylated glycoconjugates, influencing fundamental processes like blood group antigen synthesis, immune cell function and activity, cell–cell interactions, and the colonization and interaction of pathogenic microorganisms with the host. Recent studies have highlighted FUT family members, particularly FUT2 and FUT8, as potential therapeutic targets in cancers, including colorectal, breast, and lung adenocarcinoma, as well as autoimmune and inflammatory diseases [32,33].

Collectively, the FUT family constitutes the enzymatic machinery responsible for generating structurally diverse fucosylated glycans. Through differential expression patterns, linkage specificity, and cellular localization, individual FUT members establish tissue-specific fucosylation landscapes that critically influence intestinal homeostasis, immune regulation, microbial interactions, and disease progression.

#### 2.2.6. L-Fucokinase (FUK), GDP-Fucose Pyrophosphorylase (GFPP) and L-Fucokinase/GDP-Fucose Pyrophosphorylase (FKP)

FUK and GFPP are two essential enzymes that constitute the salvage pathway of GDP-L-fucose biosynthesis, a route that recycles free L-fucose derived from the turnover of glycoproteins and glycolipids or from dietary sources. FUK, first characterized from porcine kidney cytosol [34], catalyzes the initial phosphorylation of L-fucose to L-fucose-1-phosphate in an ATP-dependent manner, representing the rate-limiting and committing step of this salvage route. This enzyme is subject to strong feedback inhibition by the end-product GDP-L-fucose, indicating tight metabolic regulation [34]. FUK belongs to the GHMP kinase family and requires a divalent cation such as Mg^2+^ or Fe^2+^ for its catalytic activity [34]. Subsequently, GFPP (also designated GTP-L-fucose pyrophosphorylase, EC 2.7.7.30) catalyzes the reversible condensation of L-fucose-1-phosphate and GTP to form GDP-L-fucose, the universal fucosyl donor for all downstream fucosyltransferase reactions [35]. Human GFPP is a 66.6 kDa monomeric protein that functions primarily in the liver and kidney to salvage free fucose during the turnover of glycoproteins and glycolipids [35]. Biochemical studies utilizing a series of substrate analogues have revealed that the purine base of GTP is the major determinant of GFPP substrate specificity, particularly the hydrogen-bonding face formed by the C-6 carbonyl, N-1 nitrogen, and C-2 exocyclic amine of guanine; the hexose-1-phosphate and the ribose moiety serve as secondary determinants [36].

In certain bacterial species (such as the gut symbiont *Bacteroides fragilis*), FUK and GFPP are fused into a single bifunctional polypeptide, designated FKP, which enables the sequential conversion of L-fucose to GDP-L-fucose within a single enzyme [37,38]. This fusion protein features an N-terminal pyrophosphorylase domain and a C-terminal kinase domain, allowing efficient channeling of the intermediate L-fucose-1-phosphate [38]. In *B. fragilis*, this salvage pathway is crucial for competitive colonization in the mammalian gut, enabling the bacterium to decorate its surface with host-like fucosylated molecules that may facilitate immune tolerance [37].

Loss-of-function variants in human FUK cause a congenital disorder of glycosylation (FCSK-CDG) characterized by severe developmental delays, encephalopathy, intractable seizures, and hypotonia [39]. Patient-derived fibroblasts show significant decreases in GDP-fucose and fucose-1-phosphate levels, as well as reduced incorporation of fucose into glycoproteins [39]. In pathogenic bacteria, the salvage pathway—and FKP in particular—plays a pivotal role in host glycan foraging and mucosal colonization [37,40], highlighting this enzyme system as a promising target for anti-infective intervention. FKP has also been exploited for the chemoenzymatic synthesis of GDP-fucose and fucosylated glycans, including 2′-fucosyllactose and Lewis antigens [40]. Collectively, FUK, GFPP, and their bifunctional variant FKP constitute a conserved and functionally versatile module that fine-tunes GDP-L-fucose availability in response to metabolic demand, and their dysregulation has been implicated in immune and congenital glycosylation disorders [34,35,37,39].

#### 2.2.7. α-L-Fucosidase (FUCA)

FUCA, based on amino acid sequence similarity and catalytic mechanism, is classified into GH29, GH95, and GH151 families, with the GH29 family playing the predominant role in humans. Mammalian α-L-fucosidase adopts a single-domain barrel structure containing a conserved catalytic site [41]. Based on substrate preference, α-L-fucosidases are divided into two subfamilies: GH29A, acting on various glycosidic linkages, and GH29B, specifically catalyzing the hydrolysis of α1, 3/4-fucosidic linkages. FUCA is localized within lysosomes and primarily catalyzes the hydrolysis of α-L-fucosyl residues from the non-reducing ends of glycoconjugates via a retaining mechanism, it also possesses transglycosylation activity [42,43]. Fucosidases participate in diverse physiological and pathological processes by altering the structure of cell surface glycoconjugates. Research is exploring the therapeutic potential of fucosidase inhibitors in diseases involving dysregulated fucose metabolism, including various cancers, viral infections, and autoimmune diseases, primarily linked to fucosidase-mediated regulation of autophagy and immune cell infiltration [44,45]. Furthermore, recent studies focus on the transglycosylation function of FUCA, revealing its potential application for the in vitro production of fucosylated human milk oligosaccharides [46].

**Table 2:** Key enzymes involved in fucosylation.

Enzyme	Subcellular Localization	Function	Substrate (s)	Product (s)	Ref.
PMM2	Cytosol	Reversibly catalyzes the conversion of mannose-6-phosphate to mannose-1-phosphate	Mannose-6-phosphate	Mannose-1- phosphate	[16,17,18,19,20,21]
GDP-MP (GMPPA/GMPPB)	Cytosol	Catalyzes the formation of GDP-mannose from GTP and mannose-1-phosphate	GTP, Mannose-1-phosphate	GDP-mannose	[22,23,24]
GMDS	Cytosol	Catalyzes the conversion of GDP-D-mannose to GDP-4-keto-6-deoxymannose	GDP-D-mannose	GDP-4-keto-6- deoxymannose	[25,26,27,29]
TSTA3 (FX)	Cytosol	Bifunctional epimerase/reductase catalyzing the NADPH- dependent synthesis of GDP-L-fucose	GDP-4-keto-6-deoxymannose, NADPH	GDP-L-fucose	[22,27,30,31,47]
FUTs	FUT1–11: mainly Golgi apparatus POFUT1/2: endoplasmic reticulum	Catalyze the transfer of fucose from GDP-fucose to acceptor glycoproteins or glycolipids, forming α1,2-, α1,3/4-, α1,6-, or O-fucosidic linkages	GDP-fucose, acceptor glycans/glycoproteins/glycolipids	Fucosylated glycoconjugates	[9,11,15,32,33]
FUK	Cytosol	Phosphorylates free L-fucose to generate L-fucose-1-phosphate, using ATP as the phosphate donor	free L-fucose	L-fucose-1-phosphate	[34,35,36]
GFPP	Cytosol	Catalyzes the reversible condensation of L-fucose-1-phosphate with GTP to form GDP-L-fucose, the activated sugar donor for all fucosyltransferases.	L-fucose-1-phosphate	GDP-L-fucose	[34,35,36]
FUCA	Lysosomes	Hydrolyzes α-L- fucosyl residues from the non-reducing ends of glycoconjugates; also possesses transglycosylation activity	Glycoconjugates bearing terminal α-L-fucose	Free fucose, truncated glycoconjugates (or transglycosylation products)	[41,42,43,44,45,46]

### 2.3. Fucosylation in Cellular Processes

**Figure 2:**
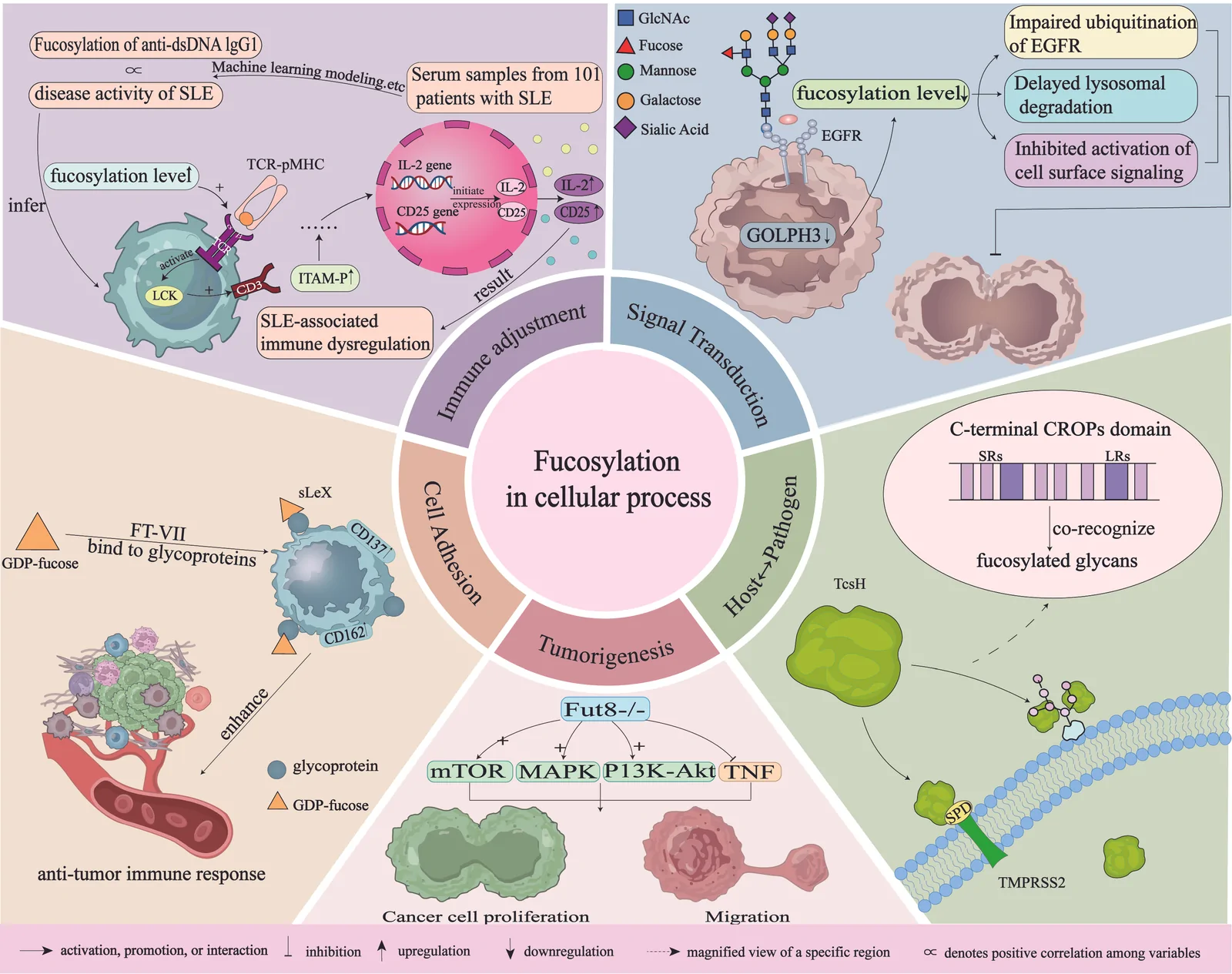
Role of fucosylation in cellular processes. The core roles and typical mechanisms of fucosylation in five major cellular processes: Host–pathogen interaction: Immune regulation: Elevated fucosylation of anti-dsDNA IgG1 in SLE patients exacerbates the disease by affecting immune cell interactions. Signal transduction: GOLPH3 knockdown in T98G glioblastoma inhibits EGFR fucosylation and blocks related signaling. Host–pathogen interaction: The TcsH toxin of *Paeniclostridium sordellii* binds to target cells by recognizing fucosylated glycans on the cell surface. Tumorigenesis: FUT8 deficiency in cervical cancer activates pro-tumor pathways and drives cancer cell proliferation and tumorigenesis. Cell adhesion: Fucosylation modifies surface molecules of CTLs, enhancing their binding to tumor vascular endothelial cells and cytotoxic efficiency. These processes provide crucial insights for investigating therapeutic strategies for intestinal-related diseases.

As a ubiquitous glycosylation modification in the biological world, fucosylation plays a key role in various cellular processes and molecular interactions. During pathogen infection, fucosylation serves as the molecular basis for host–pathogen interactions. The hemorrhagic toxin of *Paeniclostridium sordellii* recognizes and binds to multiple fucosylated glycans on the cell surface through numerous repeats in its C-terminal combined repetitive oligopeptides (CROPs) domain, thereby enabling high-affinity attachment to target cell surfaces. This strategy is consistent with the pathogenic mechanisms of various bacterial toxins such as Shiga toxin, revealing the critical role of fucosylation in the pathology of certain infections [48]. Furthermore, aberrant fucosylation is a key characteristic of tumor cell phenotypes. In pancreatic cancer stem-like cells, levels of α1,2-and α1,3/α1,4-fucosylation are elevated, accompanied by significant upregulation of mRNA expression of regulatory genes such as FUTs and GMDS. These regulatory molecules can serve as biomarkers. Detecting their expression levels can reflect cellular fucosylation levels, thereby indicating stem cell-like phenotypes in cancer cells [49]. However, deletion of the key regulatory enzyme FUT8 directly promotes cervical cancer cell proliferation, migration, and tumorigenesis in vivo by activating pro-oncogenic mTOR, MAPK and PI3K-Akt signaling pathways, while simultaneously inhibiting the TNF pathway [50]. In T98G glioblastoma cells, knockdown of Golgi phosphoprotein 3 (GOLPH3) significantly reduces the fucosylation level of epidermal growth factor receptor (EGFR). This alteration leads to impaired ubiquitination of EGFR, delayed ligand-induced endocytosis and lysosomal degradation, suppressed cell surface signaling activation, and ultimately inhibition of cell proliferation [51]. Additionally, Vamp8^−/−^ mice exhibit disrupted intestinal immune homeostasis due to reduced fucosylation of colonic mucins. This phenomenon confirms that fucosylation plays an important role in maintaining intestinal homeostasis and other cellular processes by regulating the interaction between mucins and the gut environment [52] (Figure 2).

Moreover, fucosylation can modulate immune responses by affecting interactions between immune cells and their environment. Researchers collected serum samples from 101 anti-dsDNA antibody-positive, newly diagnosed, and untreated systemic lupus erythematosus (SLE) patients. Using techniques such as quantitative glycan mass spectrometry, cluster analysis, and machine learning modeling, they analyzed the correlation between fucosylation levels and SLE disease activity index (SLEDAI) scores. The results revealed that the fucosylation of anti-dsDNA IgG1 exhibited the strongest correlation with disease activity in SLE, with its fucosylation level increasing significantly in parallel with elevated SLEDAI scores. It is hypothesized that elevated fucosylation of anti-dsDNA antibodies may alter the binding patterns of antibodies to surface receptors on immune cells such as B cells and T cells, thereby affecting the formation of immune complexes and the release of inflammatory factors, ultimately exacerbating SLE [53]. Furthermore, fucosylation regulates anti-tumor immune responses by enhancing the interaction between cytotoxic T lymphocytes (CTLs) and the tumor microenvironment. FUTs add GDP-fucose to CTLs surface glycoproteins to form the tetrasaccharide sialyl-Lewis X (sLeX). This molecular modification mediates the binding of CTLs to E-selectin and P-selectin on tumor vascular endothelial cells, promoting CTLs attachment, adhesion, and rolling, thereby enhancing CTLs homing to tumor tissues. Concurrently, a study showed that fucosylation upregulates the expression of homing molecules such as CD162 and PSGL-1, as well as costimulatory molecules like CD137 on CTLs, enhancing the cytotoxicity and improving the efficiency in killing target cells [54]. Therefore, fucosylation plays diverse and crucial roles in cellular processes, contributing to cell adhesion, signal transduction, immune regulation, tumorigenesis, and host–pathogen interactions [48,54].

## 3. Fucosylation in Intestinal Physiology

Current research highlights the intestine as a highly complex system, wherein its various components play essential roles in maintaining internal homeostasis. Tight junctions between epithelial cells, composed of proteins such as occludin and claudins, regulate the transport of small molecules and restrict the absorption of harmful substances. The intestinal mucosal barrier, primarily formed by the mucus layer secreted by goblet cells, provides crucial protection against hazardous agents. Concurrently, the diverse gut microbiota contributes significantly to nutrient metabolism and influences the progression of numerous diseases. Fucosylation, a key post-translational modification mediated by FUTs, exerts multifaceted effects on intestinal physiology. With its complex spatial distribution and functional specificity in both intestinal epithelial cells and mucins, fucosylation holds important functional relevance in preserving epithelial integrity, promoting mucin production, facilitating commensal colonization, and modulating local as well as systemic immune responses.

### 3.1. Spatial and Cell Type-Specific Distribution of Fucose Within the Intestinal Mucosa

#### 3.1.1. Regional Gradients Along the Longitudinal Axis

Fucosylation exhibits a distinct longitudinal gradient along the proximal–distal axis of the intestine. In the colon, epithelial fucose content displays regional variation, with higher levels observed proximally than distally, potentially reflecting differences in mucosal barrier function, mucus secretion, and epithelial recognition processes. Consistent with this regional pattern, mucin-associated oligosaccharides exhibit progressively reduced fucose content from the ileum to the rectum, whereas sialic acid abundance increases inversely, contributing to the establishment of an intestinal glycan and acidity gradient [55,56].

This spatial organization is further refined by region-specific expression and activity patterns of fucosyltransferases. Along the gastrointestinal tract, α1,2-fucosylation is mediated by FUT1 and FUT2, which exhibit distinct and partially overlapping spatial distributions. In the duodenum and jejunum, α1,2-fucosylation levels are relatively low and are primarily maintained by basal FUT1 expression. Notably, Paneth cells in these regions rely exclusively on FUT1-mediated α1,2-fucosylation and represent a FUT2-negative population [57]. In contrast, the ileum—the region of densest microbial colonization—exhibits robust FUT2-dependent fucosylation in villus columnar epithelial cells and goblet cells, a process precisely induced by commensal bacteria (e.g., segmented filamentous bacteria) and ILC3-derived IL-22 signaling [58,59]. In the colon, the functional territories of FUT2 and FUT1 partially overlap [60]. Peyer’s patch M cells display a distinct pattern in which α1,2-fucosylation depends exclusively on FUT1, contrasting with the FUT2-dominant phenotype of surrounding epithelial cells. These region-specific enzymatic patterns provide molecular evidence supporting the spatial organization of intestinal fucosylation [61]. These region-specific enzymatic assignments provide direct molecular evidence for the spatial organization of fucosylation.

Functional studies further demonstrate that this regional fucosylation gradient contributes directly to intestinal barrier protection. For example, L-fucose has been shown to enhance intestinal epithelial barrier integrity through FUT2-dependent fucosylation. In dextran sodium sulfate (DSS)-induced colitis mouse models and lipopolysaccharide (LPS)-stimulated Caco-2 cells, L-fucose preserves tight junction organization and improves epithelial barrier function [62,63,64]. Moreover, 2′-fucosyllactose (2′-FL) upregulates tight junction proteins TJP1 (ZO-1) and occludin via modulation of Bifidobacterium [65], and, together with 3′-sialyllactose, promotes intestinal stem cell differentiation and mucus layer integrity [66]. These findings demonstrate that the longitudinal fucosylation gradient is not merely a passive distribution pattern but an active regulatory mechanism contributing to region-specific barrier defense. However, most available studies have focused on murine ileum and colon models, and the dynamic regulation and physiological significance of segment-specific fucosylation in the human intestine remain poorly understood.

#### 3.1.2. Cell Type-Specific Fucosylation as a Marker of Differentiation and Function

Within the intestinal epithelium, fucosylation is closely associated with the differentiation and maturation of intestinal epithelial cells (IECs) as they migrate from the crypts toward the villus tips. Mature villus cells exhibit approximately three- to fourfold greater fucose incorporation than crypt cells [67]. Autoradiographic analyses following administration of ^3^H-fucose have shown that newly fucosylated glycoproteins initially appear in the trans-Golgi network, subsequently enter secretory vesicles, and are transported to the apical surface, where they become incorporated into the glycocaly [68]. Crucially, fucose-labeled glycoproteins are specifically enriched on the luminal membrane of villus cells, whereas the luminal membrane of crypt cells is almost devoid of fucosylated glycoproteins, consistent with their undifferentiated state. Within villus cells, newly synthesized fucosylated glycoproteins initially appear in the Golgi and basolateral membrane, then selectively migrate to the luminal membrane within 3–4 h in a microtubule-dependent process. Thus, fucosylation is a hallmark of IEC maturation, with the bulk of fucosylated glycoproteins synthesized predominantly in differentiated villus cells [67].

Cell type-specific fucosylation is also evident among intestinal secretory lineages. Within the crypts, Paneth cells comprise distinct FUT2-defined subpopulations: the duodenum is predominantly occupied by FUT2-negative cells, whereas the ileum contains both FUT2-negative and FUT2-positive subsets [57]. Goblet cells also exhibit a striking mosaic pattern: within colonic crypts, goblet cells secreting MUC2 with distinct α1,2-fucosylation states are arranged in an alternating, intercalated distribution [60]. Furthermore, fucose residues are key components of the neutral glycolipid GL-5 (fucosylated asialo GM1) on the IEC surface. Immunohistochemical evidence shows strong GL-5 expression in small intestinal villus epithelial cells but weaker expression in crypt epithelial cells, and GL-5 is present only in small intestinal epithelial cells (specifically in the microvillar membrane), absent in non-epithelial tissues [69,70]. Similarly, blood group A- and B-active fucosylated glycolipids are preferentially localized to epithelial cells and are largely absent from connective and muscle tissues [71]. Collectively, these findings demonstrate that fucosylation is not uniformly distributed throughout the intestinal epithelium but is spatially and cell-type specifically organized according to differentiation state and cellular function.

The functional importance of this cell-type-specific fucosylation is underscored by studies on mucin production. For example, fucosyltransferase FUT8 directly promotes secretion of mucins from the intracellular space to the extracellular environment [72]. Restoration of fucosylation via the GDP-mannose 4,6-dehydratase pathway significantly enhances MUC2 expression in intestinal adenocarcinoma cells, indicating a direct regulatory link between fucosylation and mucin biosynthesis [73]. Furthermore, in situ proximity ligation assays have revealed that MUC2 produced by goblet cells and double-goblet cell subsets across different intestinal segments displays distinct α1,2-fucosylation patterns [74]. This segmental heterogeneity is consistent with the crypt–villus differentiation gradient: villus cells incorporate 3–4 fold more fucose than crypt cells, and their fucosylated glycoproteins are Golgi-synthesized and microtubule-dependently delivered to the luminal membrane, which is virtually absent in crypt cells [67]. Given that different intestinal segments possess distinct crypt/villus ratios and migration kinetics, the MUC2 α1,2-fucosylation patterns inevitably exhibit regional specificity, directly supporting the earlier described mosaic distribution [60]. Fucosylation deficiency impairs goblet cell migration and wound-healing capacity, as goblet cells with high MUC2 output exhibit reduced migration speed due to endoplasmic reticulum stress [75,76]. Thus, the cell-type-specific allocation of fucose residues is not merely a differentiation marker but is functionally required for proper mucin biosynthesis, secretion, and epithelial repair.

These results establish fucosylation as a differentiation marker and a functional element in mucin biosynthesis and repair. However, most existing studies rely on static staining or in vitro cell lines, lacking real-time tracking of the dynamic reprogramming of fucosylation during cell fate transitions. Moreover, the physiological significance of distinct subpopulations requires validation by in vivo lineage tracing.

#### 3.1.3. Stratified Organization Within the Extracellular Mucus Layer

The spatial specificity of fucosylation extends beyond cellular compartments to the organized architecture of the secreted mucus. Once secreted, MUC2 molecules carrying or lacking α1,2-fucose residues do not intermix randomly. Instead, they assemble into immiscible, densely stratified layers within the inner colonic mucus, with the outer layer forming a loose habitat that supports commensal colonization and metabolism [60]. This hierarchical organization—from segmental macro-gradients to cellular micro-heterogeneity and layer-specific extracellular stratification—establishes a dynamic glycocalyx code that governs host–microbiota interactions at the mucosal interface. Disruption of this intricate fucosylation architecture has been recognized as a critical pathogenic factor in inflammatory bowel disease, alcoholic liver disease, and other systemic disorders [59,77].

Direct functional evidence for the stratified fucosylation pattern comes from studies on mucin barrier function. In animal models treated with botulinum neurotoxin, fucosylation of intestinal mucins in wild-type mice prevented penetration of L-PTC/A62A (a large progenitor toxin complex from *Clostridium botulinum* serotype A, strain 62A). L-PTC/A62A is a low-oral-toxicity botulinum toxin complex from serotype A strain 62A that is trapped by α1,2-fucosylated intestinal mucins and primarily enters the host through M cells), thereby blocking its entry via M cells. In contrast, FUT2-deficient mice, which lack terminal α1,2-fucose residues on mucins, exposed galactose residues that allowed toxin binding and host entry [78]. This demonstrates that the specific fucosylation state of secreted MUC2—and its layered organization—critically determines the mucus barrier’s ability to exclude pathogens. Moreover, 2′-FL and 3′-sialyllactose promote goblet cell differentiation and enhance mucus layer integrity [66], while L-fucose upregulates FUT2 and α1,2-fucosylation in intestinal stem cells, maintaining organoid growth and proliferative capacity after LPS treatment [79,80]. Collectively, these findings provide mechanistic support for a model in which region-and cell-type-specific fucosylation contributes to mucus barrier function, host–microbiota segregation, and protection against enteric toxins and pathogens.

Nevertheless, real-time characterization of this stratified organization in vivo remains technically challenging. Current evidence is derived predominantly from fixed specimens or in vitro reconstitution systems, leaving unresolved how intestinal peristalsis, mucus turnover, shedding, and microbial enzymatic degradation dynamically influence the spatial distribution of fucosylated mucins.

#### 3.1.4. Factors Influencing the Distribution of Fucosylation

Dietary factors can substantially modulate the spatial distribution of intestinal fucosylation, providing evidence that this pattern is dynamically regulated by environmental cues. A high-fat diet markedly alters glycan processing through changes in glycosyltransferase and glycosidase activities [81]. Specifically, a high-fat diet leads to a significant reduction in fucose residues in Brunner’s glands and reduces fucosylation and sulfation levels in villous goblet cells, thereby impairing the chemical defense of the mucus layer and elevating susceptibility to intestinal infections, inflammation, and tumorigenesis [82]. Conversely, a high-starch diet significantly enhances fucosylation levels in colonic epithelial cells, altering the shielding effect of fucose on other glycan chains and remodeling the glycosylation profile of the intestinal mucosa [83]. Introduction of dietary fiber (particularly cellulose and pectin) transiently enhances fucosylation of small intestinal glycoproteins, a process co-regulated by multiple enzymes but independent of short-chain fatty acid content [56,84,85]. Collectively, these dietary responses indicate that the spatial and cell type-specific distribution of fucosylation is not static but can be dynamically modulated by environmental cues.

However, most of these studies are based on short-term dietary interventions or correlational analyses and have not yet established how specific dietary components regulate fucosyltransferase activity across different cell types and intestinal segments. Furthermore, the effects of long-term dietary patterns and their interactions with the gut microbiota on intestinal fucosylation homeostasis require validation in controlled longitudinal and mechanistic studies.

### 3.2. Physiological Regulation of the Gut Microbiota by Fucosylation

#### 3.2.1. Host Fucosylation Shapes the Gut Microbial Ecosystem

In the healthy intestine, the host constructs a molecular niche centered on fucose, providing the foundation for commensal microbial colonization and metabolism. The primary executor of this process is the α1,2-fucosyltransferase encoded by FUT2, which adds fucose residues to the termini of epithelial cell surface glycans and secreted mucins, generating glycan ligands for commensal recognition and binding [86]. This provisioning mechanism does not operate constitutively and indiscriminately but is subject to tight spatiotemporal and neuroendocrine control. Key studies demonstrated that the enteric neuropeptide VIP, acting through the VIPR1 receptor on intestinal epithelial cells, activates the Erk1/2–c-Fos signaling cascade to drive FUT2 expression, thereby directly maintaining the α1,2-fucosylated niche required for colonization by beneficial symbionts such as *Bifidobacterium*, while simultaneously suppressing the overgrowth of opportunistic pathogens like *Enterococcus faecalis* [86]. This reveals a precise molecular circuit spanning from neural input to epithelial glycan output to microbial colonization. The host also engages the toll-like receptor 4(TLR4) receptor signaling pathway in the fine sculpting of the fucose niche. At the level of animal models, direct experimental evidence demonstrates a sophisticated regulatory interplay between host innate immune signalling and mucosal glycosylation. Using gene-knockout and gnotobiotic colonization mouse models, [87] established that fucosylated TLR4 (Fuc-TLR4) functions as a critical sentinel receptor which, upon sensing signals from mutualistic bacteria, directly induces FUT2 expression and thereby actively orchestrates a fucose-enriched intestinal microenvironment. This mechanism has been validated in mice as an essential prerequisite for the colonization of fucotrophic taxa such as *Bacteroides fragilis* [87]. In parallel, as reviewed by Fekete and Buret [88], mucin O-glycans, including fucosylated modifications, constitute a glycocode that serves as a key determinant of microbial community composition, metabolic output, and resistance to pathogen invasion. Nevertheless, caution is warranted when extrapolating these findings directly to humans. Current epidemiological and genetic evidence, exemplified by the association between FUT2 non-secretor status and Crohn’s disease, primarily reflects phenotypic correlations [88], whether these associations are fully mediated by the same molecular axis remains to be corroborated by human tissue-based studies or organoid models.

Exogenous fucose and fucose-rich dietary components further corroborate the universality of this niche-construction model. 2′-FL, upon colonic fermentation, modulates intestinal short-chain fatty acid (SCFA) levels and tryptophan metabolism, specifically promoting the proliferation of beneficial bacteria such as *Bifidobacterium* [89]. Notably, the combined application of 2′-FL and osteopontin (OPN, a glycoprotein that synergizes with 2′-FL to enhance the intestinal barrier and promote beneficial gut microbiota) synergistically enhances the expression of MUC2 and ZO-1 and promotes the proliferation of beneficial bacteria such as *Lactobacillus* [90], suggesting that the host may integrate multiple molecular signals to coordinately regulate the quality of the fucose niche.

#### 3.2.2. Microbial Liberation and Metabolic Conversion of Host Fucose

The gut microbiota is by no means a passive recipient of host-derived fucose. On the contrary, it actively participates in the liberation, metabolic conversion, and redistribution of this sugar through its own enzymatic machinery. Multiple commensal lineages, most prominently *Akkermansia muciniphila (A. muciniphila)*, *Bacteroides* species, and certain *Bifidobacterium* strains, encode an extensive repertoire of α-L-fucosidases (GH29 and GH95 families) capable of cleaving terminal fucose residues from mucin O-glycans, thereby modulating mucosal glycan availability and contributing to the maintenance of barrier integrity and host–microbiota homeostasis [91,92,93]. Therefore, the intestinal mucus layer serves dual functions: it forms a physical barrier while also providing a nutrient reservoir that supplies carbon and energy sources for microbial communities [92,94].

*A. muciniphila*, a mucin-degrading gut symbiont, has become an important model organism for investigating microbial utilization of host glycans [95,96]. This species promotes host mucin secretion and plays a key role in sustaining the intestinal mucus metabolic cycle and its dynamic renewal [91]. Six fucosidases, four GH29 and two GH95 family members, have been identified in *A. muciniphila* strain MucT, exhibiting functionally significant substrate specificity: GH95 enzymes preferentially hydrolyze α1,2-linked fucose residues, whereas GH29 enzymes include GH29A members with broader specificity and GH29B members that preferentially target α1,3/α1,4 linkages [93]. This enzymatic diversity means that *A. muciniphila* can process multiple types of fucose linkages in host mucins, positioning it as a hub node for fucose liberation.

Notably, *A. muciniphila* does not appear to utilize the fucose released by its own fucosidase activity. Released fucose can instead be utilized by neighboring microbial species, including clostridia, which convert it into metabolites such as butyrate, a short-chain fatty acid involved in intestinal barrier maintenance and immune regulation [91]. Similarly, human milk oligosaccharides (HMOs), which are structurally analogous to mucin O-glycans, can also be degraded by *A. muciniphila* via its homologous enzyme systems, yielding acetate, succinate, and propionate as metabolic products. Of these, acetate can be utilized by intestinal epithelial cells for energy and promotes mucin secretion, thereby contributing to the maintenance of barrier integrity [93], while propionate has been shown to enhance the development of intestinal organoids [95]. The logic of such cross-feeding—enforcing metabolic cooperation at the community level and preventing any single species from monopolizing the fucose resource—holds fundamental significance for the maintenance of gut microbial homeostasis. *A. muciniphila* is an important node within this metabolic network, but not its entirety. *Bacteroides fragilis* can cleave L-fucose units from the host mucosal surface and recycle them for the biosynthesis of its own capsular polysaccharides [80] (An elegant strategy for converting host-derived glycan building blocks into molecular camouflage). Additionally, *Bifidobacterium* species can utilize α1,2-fucosidase-modified fucosylated mucus to acquire critical adhesion sites for colonization [97]. Notably, *Escherichia coli (E. coli)* is entirely incapable of expressing α-L-fucosidase and must depend on other gut microbes to first hydrolyze free L-fucose from fucosylated glycans before uptake and utilization [94]. This hierarchical stratification of metabolic capabilities collectively constructs a structured trophic network rooted in host fucosylation.

Intestinal mucins are rich in L-fucose, whose bioavailability is largely governed by commensal-derived fucosidases, such as those produced by *Bacteroides thetaiotaomicron* [98]. This organism liberates fucose from host glycans but lacks the downstream catabolic pathways, thereby creating a public nutrient pool that can be utilised by other microbial community members [98]. *Bifidobacterium longum* subsp. *infantis* consumes fucosyllactose via a non-phosphorylated pathway, generating 1,2-propanediol and short-chain fatty acids—an exemplary consuming strategy [99]. In contrast, *Bifidobacterium kashiwanohense* tends to release fucose without significant utilisation, and this release–consumption dynamic forms the basis of intestinal fucose cycling [99].

Pathogenic bacteria have evolved diverse strategies to exploit this niche. *Enterohaemorrhagic Escherichia coli (EHEC)* senses fucose through the FusKR two-component system, which represses both LEE virulence expression and its own fucose uptake in the intestinal lumen, thereby avoiding unnecessary energy expenditure [100]. *Salmonella enterica* serovar Typhimurium, on the other hand, employs Std fimbriae to adhere to epithelial α1,2-fucose residues, promoting colonisation and inflammation, while also utilising fucose as a carbon source via the Fuc operon [98,101]. Antibiotic-induced dysbiosis weakens the competitive consumption by commensals, leading to accumulation of fucose and sialic acid released by *Bacteroides* species, which in turn drives the expansion of pathogens (*Clostridium difficile* relies predominantly on the latter sugar) [98]. The outcome of this competition is co-regulated by host FUT2 genotype, dietary composition, and microbiota structure [101]. Its dysregulation has been closely linked to inflammatory bowel disease and colorectal cancer [101]. Collectively, fucose plays a dual role as both a commensal nutrient and a pathogenic signal, and targeting this interaction network may open new avenues for the prevention and treatment of intestinal infections.

#### 3.2.3. Reverse Regulation of Host Fucosylation by Microbial Metabolites

The preceding sections have focused on how fucosylation shapes the microbial community and its metabolism. However, this regulatory relationship is bidirectional: microbial metabolites can, in turn, modulate host fucosylation levels, forming a closed feedback loop.

Recent studies have begun to reveal the molecular mechanisms underlying this reverse regulatory process. The gut commensal Odoribacter splanchnicus produces and secretes GDP-L-fucose, which promotes P-glycoprotein expression in intestinal epithelial cells through activation of the eIF4E/c-Jun signaling pathway, thereby contributing to restoration of intestinal barrier function and epithelial transport capacity [102]. The significance of this finding lies in that microbes are not merely consumers and converters of host fucose, they can themselves synthesize and secrete GDP-L-fucose and employ it as a signaling molecule to reversely regulate host–cell gene expression. This creates a complete closed loop extending from host fucosylation and microbial utilization to microbial fucose metabolites eliciting host signal responses.

Another independent regulatory pathway originates from microbial polyamine metabolism. The key metabolite N-acetylspermidine, produced by *A. muciniphila*, can inhibit PIM1(Pim-1 proto-oncogene, serine/threonine kinase), relieve its negative regulatory effect on HDAC2, and upregulate the expression of COSMC—a crucial enzyme for synthesizing the core structure of O-glycans—thereby enhancing intestinal epithelial α1,2-fucosylation levels [103]. Increased fucosylation induced by N-acetylspermidine is associated with enhanced membrane localization of tight junction proteins ZO-1 and ZO-2 and reduced secretion of the inflammatory factor C3, thereby contributing to maintenance of intestinal barrier integrity [103]. This pathway likewise provides a complete causal chain from microbial metabolite to host glycosyltransferase expression to barrier function.

Although the concept of microbial metabolite-mediated regulation of host fucosylation is emerging, current evidence remains limited to a small number of identified pathways, including GDP-L-fucose and N-acetylspermidine signaling. Given the extensive metabolic capacity of the gut microbiome and the multiple regulatory layers controlling host fucosylation—including FUT2 transcription, GDP-fucose biosynthesis, and Golgi-associated transport processes—additional microbial–host regulatory interactions are likely to exist. Furthermore, current evidence is primarily derived from single-strain studies or supplementation experiments using individual metabolites. In complex microbial ecosystems, multiple metabolites may simultaneously influence different components of the host fucosylation machinery, potentially producing synergistic, antagonistic, or redundant effects. Defining these network-level interactions represents an important future challenge for the field.

### 3.3. Fucosylation-Mediated Immune Recognition and Cross-Organ Communication

#### 3.3.1. Immune Dialogue Between Intestinal Fucosylated Glycans and C-Type Lectin Receptors

Fucosylated glycans serve not only as substrates for microbial metabolism and interfaces for microbial colonization but also as ligands for C-type lectin receptors (CLRs), thereby contributing to innate immune recognition and regulation of immune cell function within the intestinal mucosa [104,105,106,107]. As pattern recognition receptors (PRRs), CLRs constitute a critical component of the immune system for recognizing complex glycan structures, and through evolutionary adaptation, they participate in pathogen recognition, endocytosis, and antigen presentation [104,106]. Within the specific microenvironment of the intestine, Dendritic Cell-Specific ICAM-3-Grabbing Non-integrin (DC-SIGN, CD209) is the most extensively studied CLR. DC-SIGN is primarily expressed on the surface of macrophages and dendritic cells in the lamina propria and can specifically bind fucose monosaccharides as well as a spectrum of fucose-containing glycan motifs, including Lewis-X (Le^x^), Lewis-Y (Le^y^), Lewis-A (Le^a^), and Lewis-B (Le^b^) blood group antigens [104,108]. This ligand-binding profile suggests that, under physiological conditions, DC-SIGN-positive antigen-presenting cells may sense fucosylated structures present on secreted mucins and commensal bacterial surfaces, thereby integrating glycan-derived signals from the intestinal microenvironment.

HMOs provide important evidence for understanding the physiological function of DC-SIGN in intestinal immune education. DC-SIGN recognizes two classes of human milk glycans: α-fucosylated HMOs bearing Lewis antigen determinants at their non-reducing termini, and 2′-FL [107]. MUC1, a major glycoprotein in human milk, can inhibit pathogen binding to DC-SIGN on dendritic cells through its Lex moieties, suggesting a potential receptor-decoy function [107]. These observations suggest that fucosylated milk glycans may contribute to neonatal intestinal immune education through DC-SIGN-mediated interactions. However, their physiological relevance in vivo remains to be established.

The exploitation of the fucosylation DC-SIGN axis by pathogens provides, from the opposite perspective, corroborating evidence for the fundamental role of this pathway in intestinal immune homeostasis. Human pathogens such as *Helicobacter pylori* and *Schistosoma mansoni* express fucosylated glycans (e.g., Le^x^) that can bind DC-SIGN and suppress dendritic cell activation, employing a glycan molecular mimicry strategy to subvert host immunity and facilitate long-term pathogen persistence and survival [104,105,106]. Consistent with this, novel glycoantigens conjugated with the Le^x^ trisaccharide have been shown to modulate IL-12p70 expression levels and induce a pronounced Th2-polarized immune response. It is thus inferred that the interaction between pathogen-derived fucosylated glycans and DC-SIGN may disrupt the intestinal Th1/Th2 immune balance, creating an immune-privileged microenvironment favoring pathogen persistence [105,106]. This case of a physiological mechanism hijacked by pathogens paradoxically underscores the fundamental importance of the fucosylation–CLR pathway in maintaining intestinal immune homeostasis.

Although in vitro biochemical studies have firmly established the high affinity of DC-SIGN for fucosylated glycans, its in situ function in intestinal homeostasis remains largely speculative. Furthermore, beyond DC-SIGN, other intestinal C-type lectins, such as scavenger receptor C-type lectin (SRCL) and the mannose receptor, also recognize fucosylated glycan structures [105]. However, evidence regarding their cellular expression profiles, in situ ligands, and functional contributions in intestinal physiology remains limited. Addressing these questions in future research will require the development of genetic tools enabling cell-type-specific CLR deletion in the living intestine, coupled with in situ glycan ligand profiling technologies.

#### 3.3.2. Fucosylation-Dependent Gut–Liver Axis and Gut–Brain Axis Communication

The physiological impact of intestinal fucosylation is not confined to the gut; it can also participate in cross-organ communication. In this process, fucosylation itself does not act as a circulating signal. Rather, it indirectly influences distal organs by remodeling microbial metabolism.

Within the gut–liver axis, host α1,2-fucosylation can influence interactions between the intestinal microbiota and hepatic metabolism [109]. Animal studies have shown that FUT2 deficiency activates the intestinal FXR/FGF15(Farnesoid X Receptor and Fibroblast Growth Factor 15) signaling axis, alters bile acid metabolism, and alleviates steatohepatitis in a microbiota-dependent manner [109]. One possible mechanism involves fucose-derived metabolites and the enterohepatic circulation. Loss of FUT2 function alters epithelial fucosylation and may consequently reshape microbial biosynthetic activity, leading to changes in metabolites that reach the liver through the portal circulation and influence systemic immunometabolic processes [80].

Intestinal fucosylation may also contribute to gut–brain communication. Supplementation with 2′-FL can alleviate immune activation-induced behavioral abnormalities and neuroinflammation by modulating the gut microbiota, with these effects depending on microbial activity [110]. The resulting microbe–fucose–brain signaling may involve interconnected neuroendocrine, immune, and neurotransmitter pathways, thereby providing a potential mechanism for gut–brain communication [76,111].

## 4. Fucosylation Alterations in Intestinal Diseases

Alterations in fucosylation patterns represent a critical molecular hallmark in the pathogenesis and progression of various intestinal diseases, with their dysregulated expression closely linked to disease course, immune microenvironment remodeling, and clinical outcomes. This section will focus on two major intestinal disorders, IBD and CRC, to systematically elaborate the specific changes and functional implications of fucosylation within these contexts. In IBD, fucosylation plays complex and sometimes paradoxical dual roles by modulating the epithelial barrier, host-microbiota interactions, and immune cell function. In contrast, aberrant fucosylation in CRC serves as a central mechanism driving malignant progression, encompassing tumor proliferation, invasion, metastasis, stemness, and chemoresistance. Understanding these disease-specific alterations not only provides novel insights into pathological mechanisms but also establishes a theoretical foundation for developing glycosylation-based diagnostic biomarkers and targeted therapeutic strategies.

### 4.1. The Dual Roles of Fucosylation in Inflammatory Bowel Disease

**Figure 3:**
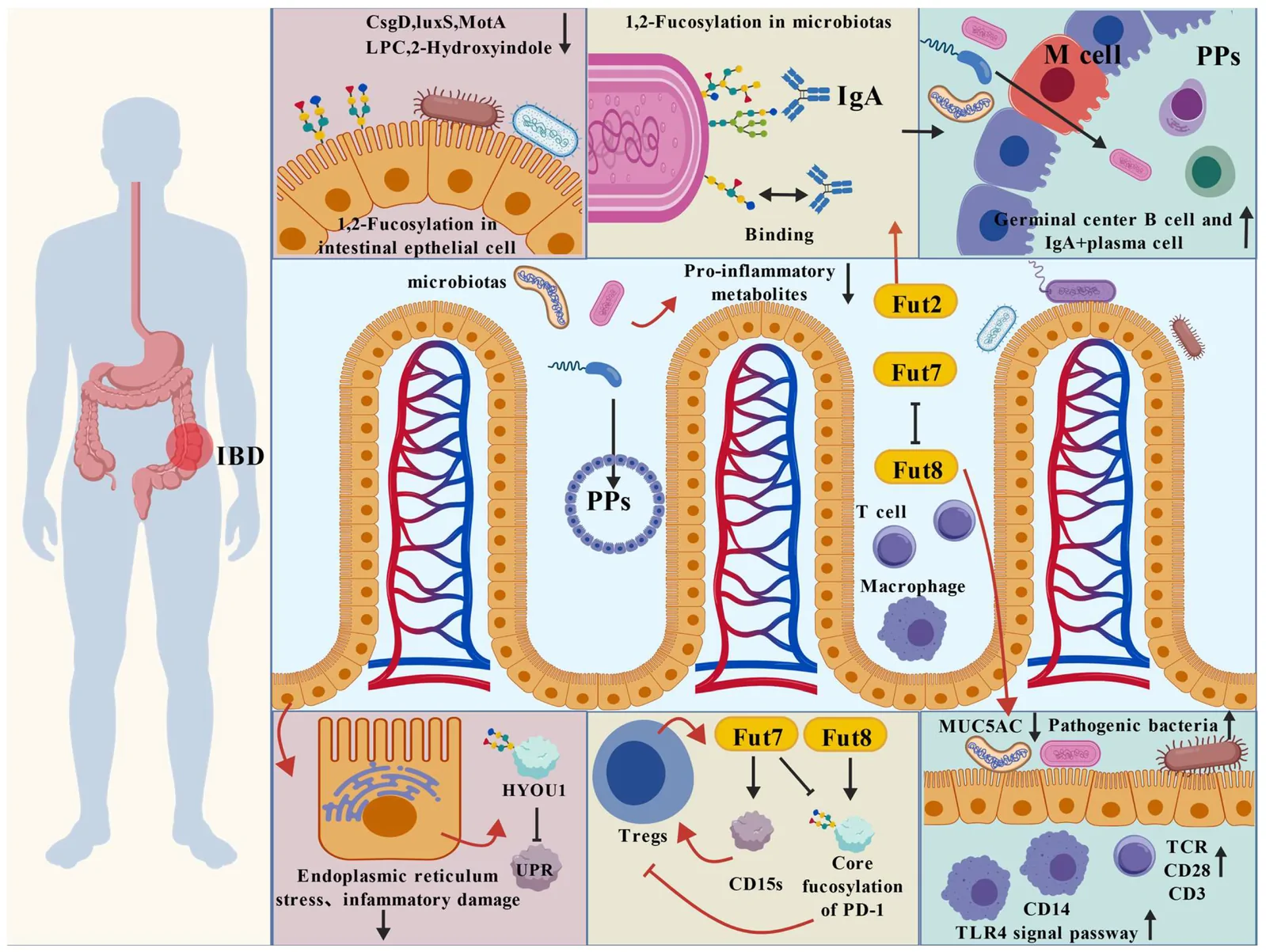
The dual roles of fucosylation in intestinal inflammation. Protective α1–2-fucosylation, primarily mediated by FUT2, sustains host-microbiota symbiosis by shaping microbial colonization, inhibiting pathogenic biofilms formation, modulating metabolites and IgA affinity, and regulating UPR. In immune cells, FUT7-generated CD15s facilitates Tregs homing. Conversely, FUT8-mediated core fucosylation often exacerbates IBD by promoting pathogenic mucus retention and enhancing macrophage and T cell inflammatory responses. Created with BioGDP.com, Dingbo Song, 2026.

IBD is a chronic relapsing intestinal inflammatory disorder driven by genetic susceptibility, environmental triggers, immune dysregulation, and gut microbiota. Fucosylation has emerged as a critical molecular node in IBD pathogenesis, yet exhibits remarkable context-dependent duality: the same enzymatic modification can exert opposing effects depending on cell type, disease stage, and local microenvironment. This section critically analyzes the multifaceted, sometimes contradictory roles of fucosylation in intestinal inflammation, emphasizing cell type–specific and stage-dependent functions of key fucosyltransferases (Figure 3, Table 3).

FUT2 catalyzes α1,2-fucosylation of epithelial glycans and plays a crucial role in regulating host–microbe balance. FUT2^−/−^ mice show heightened susceptibility to chemically induced colitis [80], yet do not develop spontaneous colitis under steady-state conditions [80,112]—indicating that FUT2 protection is disease-stage dependent, becoming functionally relevant upon inflammatory challenge rather than at homeostasis.

The protective mechanisms of FUT2 span multiple cell types. At the epithelial–microbial interface, FUT2-mediated fucosylation suppresses biofilm formation by *Salmonella Typhimurium* and *Escherichia coli* through inhibition of biofilm-related genes (csgD, luxS, motA, fliA) while enhancing swarming motility, limiting crypt occupation by invasive biofilms—an effect prominent at the early infection stage (18–24 h post-infection) [80,112]. At the host–microbiota–immune axis, FUT2-dependent epithelial fucosylation modulates bacterial surface fucosylation, essential for IgA-mediated commensal recognition [80]. FUT2 deficiency reduces bacterial surface fucosylation, impairing IgA coating, commensal translocation to Peyer’s patches, germinal center B-cell activation, and IgA^+^ plasma cell differentiation [80]. Human data confirm that FUT2 loss-of-function mutations correlate with reduced luminal fucose and bacterial fucosylation. At the intestinal stem cell (ISC) level, FUT2-mediated fucosylation of the ER chaperone HYOU1 at asparagine 862 suppresses the pro-apoptotic IRE1/TRAF2/ASK1/JNK branch of the unfolded protein response (UPR), protecting ISCs against inflammatory injury [79]. FUT2 depletion in ISCs exacerbates ER stress, mitochondrial dysfunction, impaired organoid growth, and stemness loss [79].

Despite these protective roles, the FUT2 protection narrative requires qualifications, some paradoxical. Human cohort studies reveal that fecal butyrate levels are paradoxically elevated in FUT2 non-secretors (sese) compared with secretors [113], challenging the assumption that FUT2 loss impairs microbiota through reduced SCFA substrate. Instead, metagenomic and metabolomic analyses suggest that FUT2 deficiency alters the microbial ecosystem primarily through decreased adhesion sites for adherent bacteria (e.g., *Escherichia*), allowing overgrowth of CD8^+^-inducing (*Alistipes*, *Phascolarctobacterium*) and Th17-inducing (*Erysipelotrichaceae* UCG-003) taxa [113]—a mechanism of binding-site loss rather than nutrient deprivation. Additionally, FUT2 protection is microbiota-dependent: in the absence of beneficial symbionts, or when pathogens exploit fucose as an adhesion or signaling molecule, the net outcome of fucosylation can be context-dependent [80,112]. Thus, FUT2-mediated protection emerges from a dynamic interplay between host genetics, microbial ecology, and inflammatory context.

FUT8-mediated core fucosylation (α1,6-linkage) largely exerts pro-inflammatory functions in IBD, but this generalization requires cell type–specific dissection.

In the epithelial compartment, FUT8 overexpression—elevated in inflamed distal colon of UC patients—promotes MUC5AC fiber secretion and MUC1 plasma membrane localization, generating a hyper-viscous mucus layer with enhanced resistance to shear stress [72]. This aberrant mucus, rather than protecting the epithelium, becomes a haven for pathogenic bacterial retention and invasion, exacerbating intestinal inflammation [72]. The paradox reveals a key principle: mucus mobility and renewability, not absolute quantity or mechanical strength, constitute effective defense. Mechanistically, increased MUC5AC secretion in early inflammation might serve compensatory protection, whereas sustained FUT8 overexpression in chronic disease renders the mucus maladaptively hyper-viscous. However, direct temporal evidence for this stage-dependent shift is not provided in the original study. Fut8^−/−^ mice exhibit a thinner proximal colon mucus layer with altered neutral-to-acidic mucin ratios and are protected from colitis [72]. Notably, human UC predominantly affects the distal colon, where FUT8 is elevated in patients [72]—an anatomical disparity underscoring region-specific interpretation.

In the myeloid compartment, FUT8 drives macrophage inflammation via TLR4 and TLR2 signaling. FUT8-deficient macrophages show decreased CD14 expression, impaired TLR4 signaling, and reduced inflammatory cytokine production (IL-1β, IL-10, IL-12b) [114]. Bone marrow transplantation experiments confirm that immune-cell-intrinsic FUT8 deficiency attenuates DSS-induced weight loss [114], indicating that FUT8 in hematopoietic cells promotes inflammation without necessarily altering macrophage infiltration numbers.

In the lymphoid compartment, the picture is more complex. FUT8-deficient T cells exhibit reduced core fucosylation of TCRβ, CD28, and CD3ε, impairing lipid raft trafficking, T-cell activation, and Th1/Th2 cytokine secretion, thereby alleviating colitis [115]. This supports a pro-inflammatory role of FUT8 in effector T cells. However, Fut8^−/−^ mice also show reduced Foxp3 mRNA expression and impaired TGF-β signaling [115], suggesting that FUT8 may support Treg differentiation or maintenance—an observation that cannot be definitively ascribed to Treg-intrinsic function due to the use of systemic knockout mice. This duality—FUT8 promoting effector T-cell activation while potentially supporting Treg differentiation—highlights the interpretive complexity of whole-body FUT8 deficiency in IBD models.

FUT7, the primary enzyme for CD15s synthesis in immune cells, plays a critical role in immune cell adhesion and migration. Tregs from active IBD patients show significantly reduced FUT7 expression, leading to decreased CD15s synthesis and impaired gut homing [116]. Additionally, FUT7 competes with FUT8 for GDP-fucose: high FUT7 expression inhibits FUT8-mediated core fucosylation of PD-1, promoting PD-1 ubiquitination and degradation, thereby enhancing Treg immunosuppressive capacity [116]. This dual mechanism—promoting Treg homing and enhancing Treg function—positions FUT7 as a protective fucosyltransferase in IBD.

Collectively, the evidence challenges the oversimplified protective FUT2 versus pathogenic FUT8 dichotomy. The functional outcome of fucosylation in IBD is not an intrinsic property of any single enzyme but emerges from a multi-dimensional interplay: cell type specificity, disease stage, substrate competition between FUT7 and FUT8 for limited GDP-fucose, and microbial/metabolic context. Notably, the potential role of FUT8 in Treg differentiation, suggested solely by reduced Foxp3 expression in systemic Fut8^−/−^ mice, remains mechanistically unresolved and demands Treg-specific conditional knockout studies. This complexity explains apparent contradictions in the literature and argues that therapeutic targeting of fucosylation must be cell type–specific, stage-aware, and context-sensitive, moving beyond enzyme-centric generalities toward a systems-level understanding of fucosylation in intestinal homeostasis and disease.

### 4.2. Pathogenic Mechanisms of Fucosylation in Colorectal Cancer

**Figure 4:**
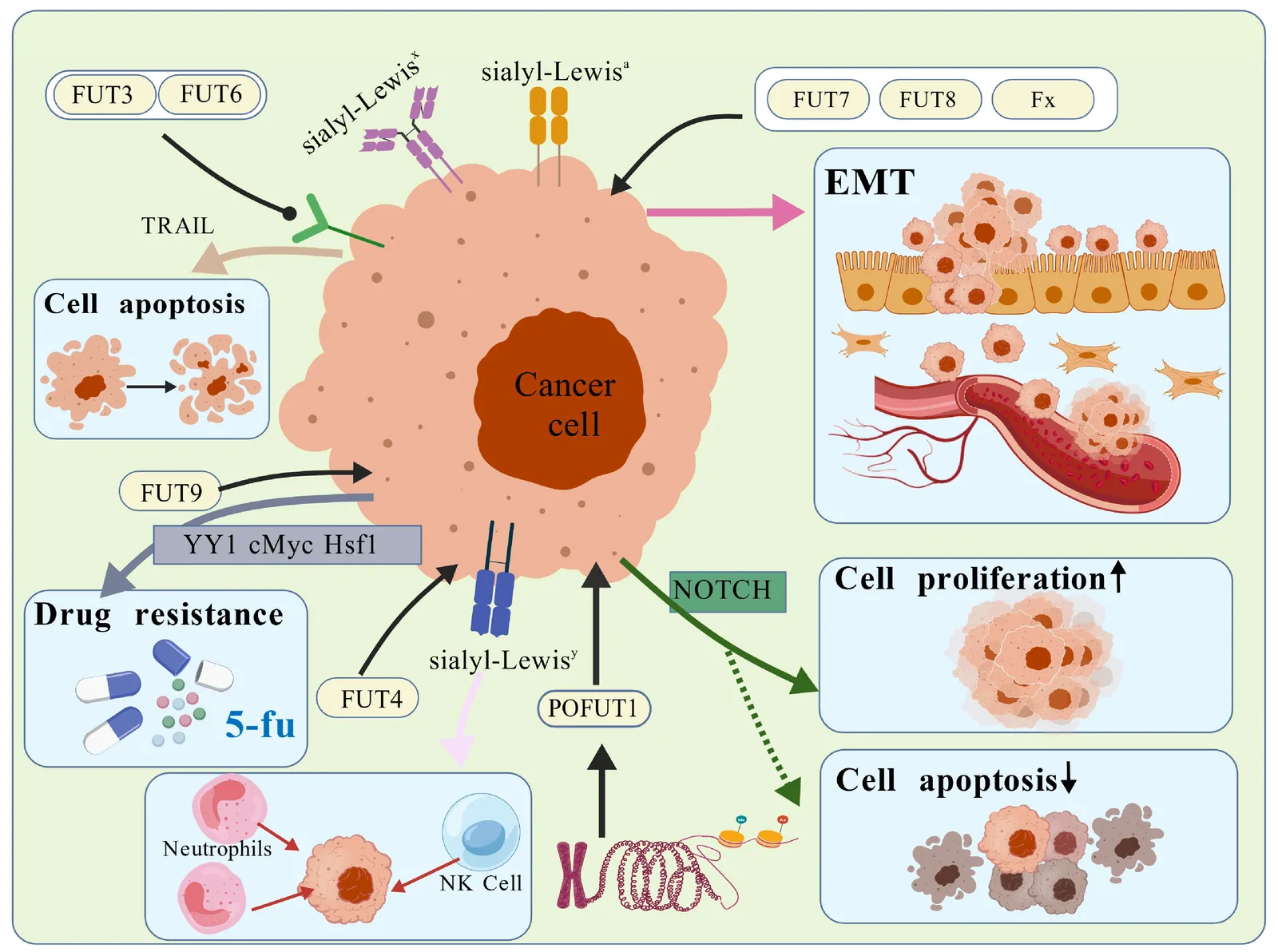
In CRC, fucosylation plays diverse roles through specific FUT enzymes. FUT3/6 increases cancer cell sensitivity to TRAIL-induced apoptosis. FUT7, FUT8, and FX enhance cancer cell adhesion to the ECM via selectin interactions. POFUT1, overexpressed due to 20q11.21 amplification, likely supports tumor growth by activating the Notch pathway, increasing proliferation and inhibiting apoptosis. FUT4 generates Lewis Y antigens, boosting immune cell cytotoxicity while suppressing tumor invasion. Additionally, FUT9 promotes a stem-like phenotype and chemoresistance by activating transcription factors like YY1 and cMyc. Created with BioGDP.com, Jinyuan Li, 2026.

CRC is the third most commonly diagnosed malignancy worldwide, and its high mortality is primarily attributable to tumor metastasis, which accounts for 90% of deaths and reduces the 5-year survival rate to below 20% [117]. Aberrant fucosylation represents a core driving mechanism of CRC malignant progression, rather than a mere epiphenomenon of the disease (Figure 4, Table 3).

Serum fucosylated N-glycan profiles in CRC patients exhibit characteristic alterations: triantennary and tetraantennary sialylated N-glycans are markedly elevated, whereas core-fucosylated biantennary N-glycans are decreased [118]. The elevation in antennary fucosylation coincides with the expression of metastasis-associated antigens such as sLe^a^/sLe^x^, while altered core fucosylation mediated by FUT8 is associated with epithelial–mesenchymal transition (EMT) and dedifferentiation, thereby promoting metastatic potential [119,120]. Moreover, the function of FUT8 in CRC is context-dependent and stage-restricted. Knockdown of FUT8 sensitizes primary SW480 cells to TRAIL-induced apoptosis, but this effect is lost in metastatic SW620 cells [121]. Furthermore, the prognostic value of FUT8 expression is modified by p53 status: in p53-negative stage II/III CRC patients, high FUT8 protein expression is significantly associated with better disease-free survival (HR = 0.28, *p* = 0.012), whereas no such association exists in p53-positive tumors [122]. These findings indicate that the biological and clinical significance of FUT8 varies with tumor genetic background and disease stage.

Different FUT isoforms exert diametrically opposed functions in CRC. FUT3 and FUT6 enhance TRAIL-induced apoptosis by fucosylating death receptor DR5, promoting ligand-independent pre-clustering of DR4 and DR5, thereby augmenting Death-Inducing Signaling Complex (DISC, the protein complex that activates caspase-8 at death receptors) assembly and caspase-8 activation [123]. In contrast, FUT2 expression is significantly downregulated in CRC tissues, and low FUT2 levels correlate with advanced pathological stages and distant metastasis. FUT2 inhibits CRC cell proliferation, migration, invasion, and EMT by catalyzing α1,2-fucosylation of target proteins such as LRP1 (Low-Density Lipoprotein Receptor-Related Protein 1, a scavenger receptor involved in endocytosis, lipid homeostasis, and signal transduction, functioning as a tumor suppressor in CRC) and MCAM (Melanoma Cell Adhesion Molecule, a transmembrane glycoprotein mediating cell adhesion and migration, whose expression correlates with tumor progression and metastasis) [33,124]. Beyond this tumor-suppressive role, FUT2-mediated α1,2-fucosylation suppresses tumor development, while its downregulation in invasive carcinomas correlates with poor prognosis [33,124]. FUT2 deficiency further promotes colorectal tumorigenesis by exacerbating intestinal inflammation [124].

FUT7 is upregulated under hypoxia and promotes the synthesis of sLex, an endothelial E-selectin ligand, thereby enhancing colon cancer cell adhesion to the endothelium—a critical step in hematogenous metastasis [125]. sLe^a^ expression positively correlates with FX enzyme levels, with high-metastatic-potential variants (e.g., SW620) expressing significantly higher FX and sLe^a^ than low-metastatic variants (e.g., SW480), and FX levels modulate cancer cell adhesion to ECM components such as fibronectin [119]. The pro-metastatic function of FUT7 is highly dependent on the hypoxic microenvironment of advanced tumors. Under normoxia, FUT7 transcription is extremely low; it is only in late-stage tumors that HIF pathway activation induces FUT7 expression, driving sLea synthesis and subsequent hematogenous dissemination [125]. Similarly, the FX enzyme shows metastasis-related differences in isogenic cell lines: SW620 (metastatic) exhibits significantly higher FX and sLea levels than SW480 (primary), leading to greatly enhanced adhesion to E-selectin [119].

FUT9 drives the reprogramming of CRC cells toward a stem-like state, functioning as a key metabolic driver in advanced CRC. FUT9 enhances resistance to 5-fluorouracil (5-FU) by activating transcription factors YY1, c-Myc, and HSF1 [126,127,128]. YY1 promotes high-grade metastasis, drug resistance, and Wnt/β-catenin pathway activation, while the gene networks regulated by YY1, c-Myc, and HSF1 are co-regulated, forming a positive feedback loop that amplifies stemness upon FUT9 induction [126,128]. However, the functional impact of FUT9 is strictly cell-subpopulation-dependent. Single-cell transcriptomic data reveal that FUT9^+^ primary CRC tumor cells represent only 0.04% of all epithelial cells, and this rare population is enriched in stemness-related genes [126]. Importantly, FUT9 knockdown enhances proliferation and migration in 2D monolayer cultures, yet suppresses tumorsphere formation and impairs 5-FU resistance in 3D tumorsphere and xenograft models [127]. FUT9 regulates the expression of Sox2 and ALDH as well as CD44 [126,127]. At the stage-specific level, FUT9 remains low in adenomas and is only upregulated in advanced cancers, where its overexpression triggers stage IV-specific metabolic reprogramming that is not recapitulated in early premalignant lesions [127]. These observations underscore that conclusions drawn solely from monolayer cultures may provide a biased view of FUT9 function.

In addition, POFUT1, frequently amplified at the 20q11.21 chromosomal region, is overexpressed in CRC and promotes cell proliferation while inhibiting apoptosis through the Notch pathway [129]. POFUT1 exhibits one of the clearest stage-specific expression patterns among fucosylation-related enzymes. Chromosomal amplification at 20q11.21 occurs in 76.02% of CRC patients and is strongly correlated with POFUT1 overexpression (*r*_s_ = 0.774). POFUT1 overexpression is already detectable at stage I (*p* < 0.001), and its high expression rate reaches 83.9% in M1(Distant metastasis)-stage patients. Moreover, POFUT1 expression is significantly higher in rectal tumors than in colonic tumors, and higher in non-mucinous adenocarcinomas than in mucinous adenocarcinomas [129]. Furthermore, Circular RNA POFUT1 (circPOFUT1) sponges miR-653-5p to transcriptionally induce WD Repeat-Containing Protein 66 (WDR66) expression and stabilizes B-cell-specific Moloney Murine Leukemia Virus Integration Site 1 (BMI1) through interaction with Insulin-like Growth Factor 2 mRNA-Binding Protein 1 (IGF2BP1), thereby promoting CRC proliferation, migration, and invasion [130].

The N-glycosylation profiles of CRC cell lines display substantial inter-line heterogeneity: among 25 cell lines, HCT116 (harboring GMDS deficiency) exhibits only 24.4% fucosylation, while most other lines show 74.4–86.4% fucosylation; moreover, CDX1/villin-positive (better-differentiated) lines are significantly associated with multi-fucosylation (*p*= 0.003) [130]. In addition, the rBC2LCN lectin shows strong binding to LS174T and DLD-1 cells but minimal affinity for HT-29, resulting in >5-fold differences in IC_50_ values for the rBC2LCN-drug conjugate [131]. Notably, serum N-glycans originate from acute-phase proteins rather than glycans shed directly from tumor tissue, which explains the poor correlation between serum and tissue glycan profiles [120].

These findings highlight the complexity of FUT family involvement in colorectal carcinogenesis and emphasize that it is critical to evaluate the specific functions and regulatory mechanisms of individual FUT isoforms in the context of different tumor genetic backgrounds (e.g., p53 mutation status, CMS molecular subtypes), disease stages (primary vs. metastatic), and microenvironmental conditions (e.g., hypoxia). However, the roles of some FUT members in colorectal cancer remain unexplored, and the molecular mechanisms underlying their context-dependent pro- or anti-tumorigenic activities are still poorly understood. Elucidating these issues will help define the functional network of the FUT family in CRC and provide a rationale for context-aware therapeutic strategies.

**Table 3:** Overview of human fucosyltransferases and their roles in intestinal health and disease in this review.

FUT	Linkage Type	Subcellular Localization	Role in IBD or CRC (Protective/Pathogenic)	Ref.
FUT1	α1,2	Golgi	IBD: Not specifically discussed CRC: Not specifically discussed	[9,61]
FUT2	α1,2	Golgi	IBD: Protective (maintains barrier, IgA homeostasis, microbial symbiosis; deficiency exacerbates colitis) CRC: Protective (inhibits EMT and metastasis via LRP1/MCAM fucosylation)	[33,80,86,124]
FUT3	α1,3/4	Golgi	IBD: Not specifically discussed CRC: Protective (enhances TRAIL-induced apoptosis via DR5 fucosylation; sensitizes resistant cells)	[123]
FUT6	α1,3/4	Golgi	IBD: Not specifically discussed CRC: Protective (enhances TRAIL sensitivity); Pathogenic (promotes angiogenesis and migration when co-expressed with FUT5)	[123,132]
FUT7	α1,3	Golgi	IBD: Protective (maintains Treg gut homing and immunosuppression; expression decreased in IBD patients) CRC: Pathogenic (hypoxia-induced upregulation promotes adhesion to endothelium) and hematogenous metastasis)	[116,125,133]
FUT8	α1,6	Golgi	IBD: Pathogenic (promotes aberrant MUC5ac secretion, enhances macrophage/T-cell inflammatory responses) CRC: Pathogenic (promotes EMT, dedifferentiation, and metastatic potential)	[9,72,114,115,120]
FUT9	α1,3	Golgi	IBD: Not specifically discussed CRC: Pathogenic (reprograms cells toward stem-like phenotype; promotes chemoresistance via YY1/c-Myc/HSF1)	[126,127,128]
POFUT1	O-fucose	ER	IBD: Not specifically discussed CRC: Pathogenic (frequently amplified at 20q11.21; promotes proliferation and inhibits apoptosis via Notch; circPOFUT1 drives metastasis)	[11,15,129,130]

## 5. Targeted Fucosylation as a Diagnostic Approach in Intestinal Diseases

### 5.1. Targeted Fucosylation as a Diagnostic Approach for IBD

Fucosylation patterns have been demonstrated as potential biomarkers for the diagnosis of IBD, assessment of disease activity, and prediction of treatment response [134,135]. Clinical data indicate significant downregulation of FUT2 expression and reduced α-1,2-fucosylation levels in the colonic tissues of patients with UC and CD. Similarly, FUT2 protein levels and overall fucosylation are markedly decreased in the colons of DSS-induced colitis mouse models. These findings highlight FUT2 deficiency and aberrant fucosylation in both IBD patients and experimental models, providing robust support for the potential use of fucosylation patterns as diagnostic or prognostic biomarkers for IBD [136].

The FUT2 and FUT3 genes are located on chromosomes 19q13 and 19p13, respectively, within chromosomal regions (IBD6) associated with IBD susceptibility [137]. In recent years, multiple studies have confirmed that several single-nucleotide polymorphisms in the FUT2 gene are associated with susceptibility to inflammatory bowel diseases, including CD and UC [138]. The non-secretor phenotype resulting from FUT2 mutations reshapes the community composition and diversity of the gut microbiota. Microbial dysbiosis increases the risk of various diseases, such as infections, metabolic disorders, and IBD [139]. Mechanistically, it has been demonstrated that intestinal epithelial Fut2 deficiency modulates the gut microbiota, subsequently increasing the production of LPC. LPC, in turn, regulates IBD susceptibility by promoting the release of pro-inflammatory cytokines and exacerbating epithelial barrier damage, thereby aggravating colitis [136].

Furthermore, the association between FUT2 and IBD susceptibility has been extensively reported across different populations, yet considerable heterogeneity exists among study findings [140]. The association between the FUT2 rs601338 locus and IBD varies markedly depending on the study population: in Caucasians, the non-secretor status conferred by this locus has been shown to be associated only with CD risk, not with UC. Conversely, data from a Finnish cohort yielded the opposite conclusion, where carriage of the wild-type GG genotype at rs601338 increased the probability of UC [138]. In China, FUT2 rs1047781 and rs601338 have been identified as UC-associated mutation sites. Some domestic studies have found that FUT2 mutations (rs281377, rs601338) may be associated with UC susceptibility in the Han population of Xinjiang, whereas FUT2 mutations (rs1047781, rs601338) are associated with UC susceptibility in the Uyghur population [137]. These observations suggest that the association between FUT2 polymorphisms and IBD susceptibility may exhibit regional and ethnic differences.

To date, global studies on the association between FUT3 polymorphisms and IBD remain relatively scarce. One relevant study in the Chinese population reported that the dominant, recessive, and co-dominant genetic models, as well as allele frequencies of the FUT3 rs3745635 locus, are all correlated with IBD. Mutations at the FUT3 rs3745635 locus lead to aberrant expression of intestinal Lewis antigens, which in turn induces gut microbiota dysbiosis, mucosal immune derangement, and intestinal inflammatory injury, thereby modifying IBD susceptibility [141]. In another study of Chinese UC patients, the results showed that, compared with patients with extensive colitis, those with distal colitis carried a higher proportion of mutant alleles and genotypes of FUT3 (rs28362459, rs3745635). These findings suggest that FUT3 mutations are not only associated with UC pathogenesis but may also influence the distribution of lesion sites [138]. From a clinical stratification perspective, genotyping of FUT2 and FUT3 in patients could facilitate screening of high-risk IBD populations and prediction of disease subtypes, demonstrating potential for clinical diagnostic application.

Moreover, characteristic abnormalities in fucosylation are observed in serum and tissue samples from IBD patients. A research team developed an ELISA based on the 10–7G monoclonal antibody to quantify serum levels of fucosylated haptoglobin (Fuc-Hpt), measured as the 10–7G value. The production of Fuc-HPT by immune cells is associated with local immune conditions, such as those linked to cancer and inflammation. This assay was applied to clinical samples from 110 UC patients, 45 CD patients, 11 patients with acute enteritis (AE), and healthy volunteers (HVs). The results demonstrated significantly higher serum 10–7G values in IBD patients compared to HVs (*p* < 0.001), confirming elevated Fuc-Hpt levels in the serum of IBD patients. The sensitivity of this marker notably surpassed that of traditional inflammatory indicators such as C-reactive protein (CRP), suggesting it as a reliable, non-invasive diagnostic tool for IBD [142]. Additionally, fucosylation profiling of IgG antibodies revealed distinctive glycosylation features within the Fc region in IBD patients. Notably, these included galactose deficiency and increased fucosylation. These alterations exhibited disease subtype specificity, with elevated fucosylation observed in CD patients and decreased levels in UC patients. Such characteristics can facilitate differentiation between disease subtypes and provide insights into disease activity [134]. In summary, fucosylation patterns hold significant potential as biomarkers for IBD diagnosis, subtype differentiation, and activity assessment. While existing evidence supports their utility, further validation is essential before their widespread clinical application.

### 5.2. Targeted Fucosylation as a Diagnostic Approach for CRC

Aberrant glycosylation is a critical hallmark of cancer [131,143] (Table 4). In CRC, multilayered alterations in fucosylation, ranging from glycan structures to the expression of fucosylation-related enzymes, provide multiple candidate biomarkers for clinical development [118].

CRC tissues exhibit increased fucosylation of glycosphingolipid (GSL) glycans, accompanied by reductions in acetylation, sulfation, and disialylated gangliosides [144,145]. In serum, the characteristic CRC fucosylation profile—characterized by elevated triantennary and tetraantennary sialylated N-glycans and decreased core-fucosylated biantennary N-glycans—shows diagnostic potential, although it remains to be validated in large prospective cohorts [118].

Fucosylated variants of carcinoembryonic antigen (CEA), particularly its N-glycosylated forms, show superior specificity in CRC screening compared with conventional CEA [146,147]. The degree of α1-acid glycoprotein (AGP) fucosylation correlates with poor CRC prognosis, and elevated relative abundance of fucosylated AGP in serum serves as a prognostic indicator [148]. Notably, serum Fuc-Hpt levels are significantly elevated in patients with CRC and are associated with distant metastasis and advanced clinical stage; Fuc-Hpt-positive patients have significantly lower overall survival and recurrence-free survival than Fuc-Hpt-negative patients. Fuc-Hpt shows greater sensitivity than CEA for detecting distant metastasis, while their combination improves prognostic performance [149]. Importantly, Fuc-Hpt is not produced by CRC cells themselves but is synthesized by hepatocytes and lymphocytes within the tumor microenvironment, suggesting that it reflects a systemic host response to the tumor [149].

Fucosylated antigens CA19-9 and SLe^x^ are highly expressed in CRC patients and associated with unfavorable prognosis [146]. Heavily fucosylated glycans (HFGs) are significantly overexpressed in CRC tumor tissues with negligible expression in normal tissues, demonstrating high tumor specificity and potential as diagnostic targets [117].

FUT2, FUT3, and FUT4 are aberrantly expressed in CRC, with their expression levels correlating with multiple aspects of cancer pathology, and may offer ancillary diagnostic and prognostic value [147,150]. A prognostic model incorporating YAP1, SREBP-1, FUT2, and FAT2 constructed using LASSO regression provides a new molecular tool for CRC prognostic assessment [151].

The current body of evidence for fucosylation-based CRC biomarkers derives predominantly from retrospective studies and small cohorts. Most individual markers (e.g., AGP, Fuc-Hpt, HFGs) have been validated in only single or few studies and lack multi-center, prospective clinical utility assessments. Moreover, the sensitivity and specificity of any single marker alone are generally insufficient to meet the demands of population screening. Diagnostic models that integrate multiple fucosylation markers with existing clinical parameters represent a key direction for improving clinical applicability (Table 4).

**Table 4:** Diagnostic applications of fucosylation-related biomarkers in intestinal diseases.

Disease	Biomarker Type	Specific Biomarker	Sample	Level of Evidence	Clinical Significance	Ref.
IBD	Tissue biomarker	FUT2 expression α-1,2-fucosylation	Colonic tissue	Clinical research; Animal models	Significantly downregulated in UC and CD patients, consistent with DSS colitis model, suggesting diagnostic potential	[103]
	Serum biomarker	Fuc-Hpt	Serum	Clinical cohorts	Detected by 10–7G mAb ELISA, significantly elevated in IBD patients, superior diagnostic sensitivity to CRP	[133]
	Molecular biomarker	fucosylation profiling of IgG antibodies	Serum	Clinical research	Increased in CD, decreased in UC, useful for subtype differentiation and disease activity assessment	[113]
CRC	Glycolipid biomarker	fucosylation of GSL	Tumor tissue	In vitro	Positively correlated with tumor differentiation, accompanied by decreased acetylation/sulfation	[115]
	Glycoprotein biomarker	fucosylated variants of CEA	Serum/tissue	Clinical research	More specific than conventional CEA, applicable for CRC screening	[117]
	Serum glycoprotein	fucosylation of AGP	Serum	Clinical research	Branching degree and fucosylation level negatively correlated with prognosis	[129]
	Tumor antigen	fucosylated antigen CA19-9 or SLe^x^	Serum/tissue	Clinical research	Associated with poor prognosis	[146]

## 6. The Therapeutic Significance of Fucosylation in Intestinal Diseases

**Figure 5:**
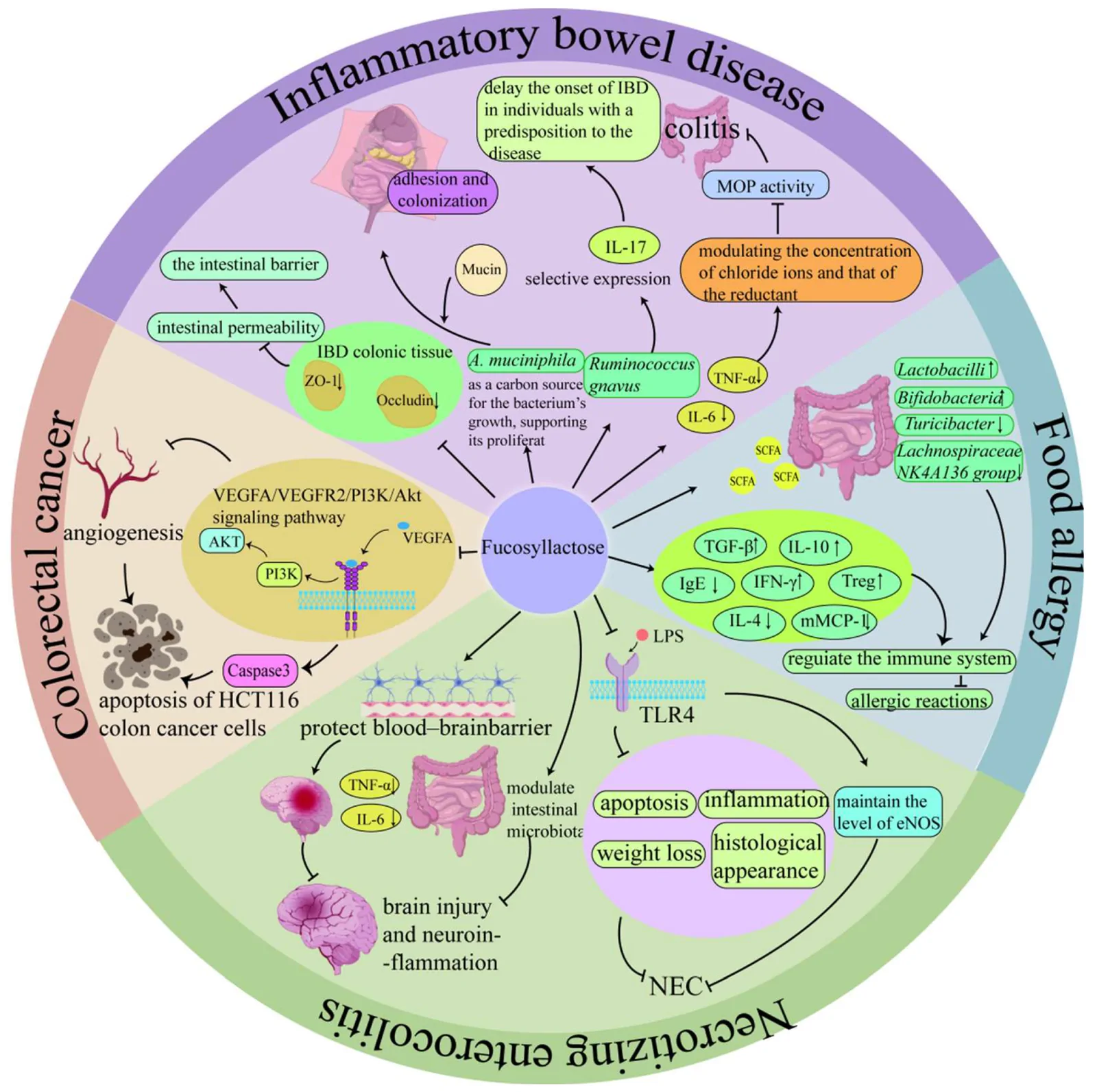
Overview of the regulatory mechanisms of fucosyllactose in IBD, CRC, necrotizing enterocolitis (NEC), and food allergy (FA) disorders. Fucosyllactose exerts its biological effects through multiple pathways: In IBD, it delays disease onset by preserving intestinal barrier integrity, modulating gut microbiota, and regulating inflammatory cytokines, chloride ion concentrations, and reducing substrate levels. In CRC, it inhibits kinase phosphorylation in the VEGFA/VEGFR2/PI3K/Akt signaling pathway, activates caspase-mediated apoptosis, and suppresses angiogenesis. In NEC, fucosyllactose mitigates brain injury and intestinal inflammation by inhibiting the TLR4/LPS pathway, protecting the blood–brain barrier, and downregulating inflammatory factors such as IL-6 and TNF-α. Concurrently, fucosyllactose modulates the immune system and alleviates FA by regulating cytokines and Tregs, as well as influencing gut microbiota and SCFA production.

Recent advancements highlight the pivotal role of fucosylation regulation in a range of inflammatory and malignant diseases. Modulating fucosylation pathways through dietary, microbial, or pharmacological interventions could present innovative and effective therapeutic strategies for various intestinal diseases, including IBD, CRC, and other intestinal diseases (Figure 5, Table 5).

### 6.1. The Therapeutic Significance of Fucosylation for IBD

In recent years, fucosylation regulation has become an emerging strategy for the treatment of IBD, particularly in the area of exogenous supplementation of fucoidan and related sugars. Recent studies have increasingly investigated the benefits of exogenously supplemented L-fucose, which primarily exerts its effects via the FUT2 pathway by supporting glycan chain biosynthesis. This intervention has demonstrated potential in mitigating damage to the intestinal mucus barrier in murine models and in maintaining the delicate balance between pathogens and the host microbiota. Specifically, exogenously supplied L-fucose enhances bacterial surface α1–2 fucosylation, which can lead to a reduction in the production of pro-inflammatory metabolites (such as D-glycero-D-manno-heptose 1-phosphate and homocysteine thiolactone [HT]) by gut microbes (e.g., *Fusobacterium nucleatum* [Fn]). This decrease in inflammatory mediators contributes to reduced intestinal inflammation, preservation of tight junction integrity in epithelial cells, alleviation of autophagy blockade, and decreased cell apoptosis [136]. Furthermore, research has shown that L-fucose supplementation activates the aryl hydrocarbon receptor (AHR), a key regulator in mucosal immune responses. Activation of AHR facilitates the release of IL-22 from CD4^+^ T cells and lamina propria mononuclear cells (LPMCs), which collectively promote tissue repair. Notably, this pathway has been linked to the repair of TNF-α-induced damage in intestinal organoids, highlighting a promising therapeutic avenue for inflammatory intestinal diseases [152].

Recent evidence has found that HMOs significantly influence gut microbiota composition and the regulation of IBD. Under physiological conditions, key HMOs such as 3′-fucosyllactose (3′-FL) and 2′-FL, are synthesized via the transglycosylase activity of α-L-fucosidases. Research indicates that HMOs, particularly 2′-Fucosyllactose, can boost the production of SCFAs, specifically acetate and propionate, in the intestinal lumen through fermentation. This process indirectly modulates immune responses and supports intestinal barrier integrity [153]. Post-lactation oral administration of 2′-FL induces beneficial shifts in the gut microbial community in the context of IBD, markedly promoting the proliferation of the commensal bacterium *Ruminococcus gnavus*. This results in selective expression of the cytokine IL-17 and contributes to delaying the onset of disease in individuals with a predisposition to IBD [154]. Furthermore, 2′-FL has been shown to downregulate the expression of acute-phase proteins, including haptoglobin and serpina3n, as well as oxidative stress markers like Cyp2e1. Simultaneously, it promotes the expression of antimicrobial peptides such as Reg3b and Reg3g, contributing to the restoration of gut microbiota homeostasis. Additionally, 2′-FL inhibits STAT3 activation by modulating its palmitoylation and depalmitoylation, subsequently reducing the inflammatory response [155,156]. Similarly, 3′-FL and 2′-FL can exert synergistic anti-inflammatory effects in IBD, reversing IL-6-induced impairment of intestinal barrier function [157]. Significantly inhibited the DSS-induced decline in the expression levels of TJP1 in colonic tissues of IBD models, thereby reducing intestinal permeability and ameliorating gut barrier dysfunction. Furthermore, 2′-FL and 3′-FL markedly decreased serum levels of pro-inflammatory cytokines, including IL-6 and TNF-α, modulated chloride ion concentrations and reducing substrate levels to alleviate oxidative stress, and suppressed myeloperoxidase (MPO) activity. Collectively, these anti-inflammatory and antioxidant effects contributed to the attenuation of DSS-induced colitis [157]. 2′-FL acts as a carbon source for *A. muciniphila*, sustaining its growth and facilitating its colonization within the murine gut, while also stimulating mucin production, which in turn enhances the adhesion of this bacterium to intestinal epithelial cells [158]. In summary, these studies suggest that regulating fucosylation through dietary or microbial metabolic interventions can provide new therapeutic approaches for mucosal repair and immune homeostasis recovery in IBD.

### 6.2. The Therapeutic Significance of Fucosylation for CRC

Given the central driving role of fucosylation in CRC malignant progression, targeting multiple nodes of the fucosylation machinery is being developed as a therapeutic approach. Current therapeutic interventions can be categorized into three main directions.

Inhibition of fucose biosynthesis. GDP-fucose synthase (TSTA3/FX) catalyzes a rate-limiting step in fucosylation, and its inhibition can block the substrate supply for multiple FUTs upstream. The flavonoid eriodictyol suppresses the clonogenic formation, invasion, and migration capabilities of CRC cells by downregulating TSTA3 expression; combining eriodictyol with the fucosylation inhibitor 2-F-FUC further enhances its anti-tumor effects [145]. Additionally, LncGMDS-AS1 (Long non-coding RNA GMDS Antisense 1) is highly expressed in CRC cells, and downregulating its expression via genome-editing approaches such as CRISPR-Cas9 exerts anti-tumor activity [159]. The advantage of this strategy lies in its broad-spectrum nature—inhibiting GDP-fucose synthesis simultaneously affects multiple pro-oncogenic fucosylation pathways. However, its risk equally lies in this broad spectrum—systemic suppression of fucosylation may compromise physiological fucosylation functions in normal tissues.

Targeting specific fucosyltransferases is also a strategy for CRC therapy. FUT8 has emerged as the most prominent small-molecule target owing to its central role in multiple pro-oncogenic pathways. Novel FUT8 inhibitors, such as FDW028 and N-(4-aminophenethyl)-4-(5-(3-(trifluoromethyl)phenyl)-1H-pyrazol-3-yl)benzamide, display potent anti-proliferative effects on CRC cell lines including SW480 and HCT-8, with favorable safety profiles [160,161]. Afucosylated therapeutic antibodies (e.g., mogamulizumab and obinutuzumab), already approved for hematological malignancies, warrant attention for their potential in CRC therapy; combining FUT8 inhibitors with afucosylated antibodies or immune checkpoint inhibitors may yield synergistic anti-tumor effects [162]. Targeting strategies for other FUTs are also under exploration: FUT1/FUT2 are considered to exert tumor-suppressive functions, and appropriately enhancing their activity may inhibit metastasis [163], while FUT3/4/5/6 and POFUT1 primarily act as pro-oncogenic factors and can be intervened with the small-molecule inhibitor SGN-2FF, regulatory miRNAs, or direct enzymatic inhibition [132,164]. These strategies have demonstrated potential in preclinical models to suppress tumor proliferation and invasion, reverse drug resistance, and overcome PD-L1-mediated immune evasion [165].

In addition, L-fucose supplementation activates the fucose salvage pathway, restoring TRAIL sensitivity in DR5-resistant cells by 5–10-fold [123]. Anti-HFG monoclonal antibodies inhibit CRC cell growth via complement-dependent cytotoxicity (CDC) and can synergistically enhance cytotoxic effects when combined with chemotherapeutic agents; the low expression of HFGs in normal tissues suggests potentially lower on-target, off-tumor toxicity [117]. Additionally, combined treatment with oxytocin and L-fucose promotes MUC2 maturation via B3GNT7-mediated fucosylation, reducing tumor burden in colitis-associated cancer (CAC) models [166]. Angiogenesis is essential for tumor growth and metastasis. By regulating the VEGFA/VEGFR2/PI3K/Akt pathway, 2′-FL suppresses angiogenesis and activates the caspase-3-mediated apoptotic pathway to induce tumor cell apoptosis. Furthermore, HCT116 cells exhibit unique drug sensitivity to 2′-FL, providing a new research direction for nutritional targeted intervention in colorectal cancer [167].

### 6.3. The Therapeutic Significance of Fucosylation for Other Diseases

Beyond IBD and CRC, targeting fucosylation and its related functional components also demonstrates significant therapeutic potential in other diseases, such as necrotizing enterocolitis and food allergy. NEC is a common and often fatal intestinal disease in preterm infants. It occurs in the context of gut bacterial colonization, formula feeding, and activation of the innate immune receptor TLR4, with limited treatment options [168,169]. HMOs have been shown to possess key immunomodulatory effects and can reduce the risk of NEC. Breastfed infants have a 6–10 times lower risk of developing NEC compared to formula-fed infants. Studies indicate that the neutral HMO fraction containing high concentrations of 2′-FL improves survival rates and reduces pathological scores in NEC models [169]. The safety of 2′-FL has been established in preclinical and clinical studies, and it was approved as a food additive in infant formula in China in October 2023 [158,169]. One of the most devastating complications of NEC is the development of NEC-induced brain injury. Administration of 2′-FL or 6′-SL can interfere with the pro-inflammatory gut–brain axis through multiple pathways. These include protecting the blood–brain barrier, reducing IL-6 and TNF-α in the brains of NEC mice, and modulating the gut microbiota to foster a potentially anti-inflammatory microbiome. This significantly alleviates NEC-induced neuroinflammation and brain injury, reverses myelination loss in the corpus callosum and midbrain of neonatal mice, and prevents cognitive impairment [170]. It has been hypothesized that 2′-FL may act as a structure-specific prebiotic, helping to construct an ideal gut microbiome or prevent NEC-associated dysbiosis [169]. In NEC, activation of intestinal epithelial TLR4 can induce epithelial cell apoptosis. The potential interaction of 2′-FL with the LPS-binding site of TLR4 suggests that it may prevent and mitigate NEC by inhibiting TLR4 signaling and maintaining Endothelial Nitric Oxide Synthase (eNOS, an enzyme that produces nitric oxide in vascular endothelial cells) levels, thereby reducing apoptosis and inflammation, and improving histological appearance [168,171].

Food allergy (FA) is a widespread global concern, affecting up to 10% of the population in developed countries, with approximately 250 million patients worldwide. HMOs, such as 2′-FL and 3′-FL, exhibit significant therapeutic and preventive effects against FA. Mechanistically, they alleviate allergic symptoms by modulating immune responses, reducing allergic factors (specific IgE, mMCP-1, IL-4) and Th2/Th1 imbalance [119], increasing anti-inflammatory factors (IFN-γ, IL-10, TGF-β) and Treg cell levels [125], enhancing intestinal barrier integrity, and modulating the gut microbiota by promoting beneficial bacteria like *Lactobacillus* and *Bifidobacterium* while reducing pathogenic and allergy-associated taxa. Notably, supplementation with 2′-FL during gestation or sensitization periods can exert protective effects by improving maternal-fetal immune competence and mitigating allergic responses. In comparative studies, the efficacy of 2′-FL and 3′-FL in FA treatment is even superior to that of GOS [172]. The successful application of fucosylated human milk *oligosaccharides* in NEC and FA highlights the broad prospects of glycan-based nutritional interventions for preventing and treating critical early-life diseases, laying a solid theoretical foundation for their clinical translation and product development.

**Table 5:** Therapeutic strategies targeting fucosylation in intestinal diseases.

Disease	Therapeutic Strategy	Intervention	Mechanism of Action	Level of Evidence	Therapeutic Effect	Ref.
IBD	Exogenous fucose supplementation	L-fouse	Promoting glycan synthesis via FUT2 pathway; activating AHR/IL-22 signaling	Animal models; in vitro	Repairing mucus barrier; inhibiting pro-inflammatory metabolites; promoting tissue repair	[103,119]
	Human milk oligosaccharides	2′-FL,3′-FL	Producing SCFAs via gut microbiota fermentation; inhibiting STAT3 activation; promoting antimicrobial peptides Reg3b/Reg3g expression	Animal models; in vitro	Immunomodulation; maintaining barrier integrity; anti-inflammation	[120,123,124,125]
CRC	Fucose synthase inhibitor	Eriodictyol,2-F-FUC	Inhibiting TSTA3 expression, reducing fucosylation modification	Animal models; in vitro	Inhibiting colony formation, invasion, and migration	[127,128]
	Fucosyltransferase inhibitor	FDW028 and N-(4-Aminophenethyl)-4-(5-(3-(trifluoromethyl)phenyl)-1H-pyrazol-3-yl)benzamide	Inhibiting core fucosylation	Animal models; in vitro	Inhibiting SW480/HCT-8 cell proliferation; favorable safety profile	[134,166]
NEC	Human milk oligosaccharides	2′-FL, 3′-SL	Inhibiting TLR4/LPS signaling; protecting blood–brain barrier; modulating microbiota	Animal models; in vitro	Reducing mortality; alleviating brain injury; improving cognitive function	[131,144,147,148]
FA	Human milk oligosaccharides	2′-FL,3′-FL	Modulating Th2/Th1 balance; increasing Tregs; promoting beneficial bacteria (*Lactobacillus, Bifidobacterium*)	Animal models; in vitro	Alleviating allergic symptoms; superior to GOS	[150,173,174]

## 7. Conclusions

Fucosylation is a critical post-translational modification that profoundly influences intestinal homeostasis and serves as a key regulatory node governing mucosal integrity, host–microbiota symbiosis, and immune function. Current evidence demonstrates that balanced fucosylation is essential for maintaining the intestinal barrier and orchestrating protective responses. Conversely, dysregulated fucosylation constitutes a hallmark of intestinal pathology, triggering chronic inflammation, disrupting microbial ecology, and promoting tumor progression and metastasis, thereby playing a pivotal role in the pathogenesis of intestinal diseases such as inflammatory bowel disease (IBD) and colorectal cancer (CRC). Nevertheless, significant gaps and challenges remain in translating these mechanistic insights into clinical applications. Despite these obstacles, the continuously evolving understanding of specific fucosyltransferases, biosynthetic enzymes, and fucosylated glycans continues to reveal their considerable potential as diagnostic biomarkers and therapeutic targets.

Future investigations can leverage cutting-edge technologies, including single-cell and spatial glycomics, in situ tracing of enzymatic activity, conditional gene knockout models, and intestinal organoid systems to systematically delineate the spatiotemporal distribution patterns of fucosylation across distinct intestinal segments, developmental stages, and disease states. Such approaches will further elucidate the precise division of labor and coordinated regulatory networks among FUT isoforms, FUCA, and key biosynthetic enzymes in modulating mucin O-glycan modification, intestinal epithelial differentiation, and microbial colonization, thereby refining the molecular framework through which fucosylation maintains intestinal homeostasis. Building upon these mechanistic foundations, future research should prioritize the development of non-invasive diagnostic models based on fucosylation profiles derived from serum, feces, and other biofluids, and accelerate multi-center, large-sample clinical validation of biomarkers such as Fuc-Hpt, fucosylated immunoglobulins, and sialyl Lewis antigens, with the aim of improving the accuracy and clinical utility of early screening, disease subtyping, and prognostic assessment in IBD and CRC. Concurrently, efforts should be directed toward accelerating the development of isoform-selective fucosyltransferase inhibitors, fucosidase modulators, and antibody-based therapeutics targeting fucosylated epitopes. These pharmacological strategies can be integrated with safe and translatable interventions, including exogenous L-fucose supplementation, nutritional intervention with fucosylated human milk oligosaccharides, and engineered probiotic enzyme delivery systems to construct clinically applicable, targeted therapeutic regimens for intestinal diseases. Furthermore, an in-depth exploration of the signaling and metabolic regulatory mechanisms mediated by fucosylation within the gut–microbiota–brain axis and the gut–microbiota–liver axis is warranted, which will help uncover the association between fucosylation dysregulation and systemic diseases, thereby expanding the research frontiers and translational potential of fucosylation in the diagnosis and treatment of systemic disorders.

In conclusion, fucosylation represents a critical regulatory nexus linking intestinal homeostasis to disease initiation and progression. Through systematic advancements in mechanistic dissection, targeted tool development, diagnostic biomarker validation, and translational intervention strategies, fucosylation holds considerable promise for delivering novel, reliable diagnostic biomarkers and personalized therapeutic approaches for intestinal diseases such as IBD and CRC. These endeavors are poised to propel the field of glycosylation regulation from fundamental research toward clinical application, providing new theoretical foundations and technological pathways for the precision prevention and treatment of intestinal disorders.

## Figures and Tables

**Table 1:** Key metabolites involved in fucosylation.

Metabolite	Molecular Formula	Chemical Structure	Upstream Enzyme	Downstream Enzyme
D-Glucose (Glc)	C6H12O6	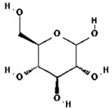	—	Hexokinase/Glucokinase
D-glucose 6-phosphate (Glc-6-P)	C6H13O9P	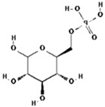	Hexokinase/Glucokinase	Glucose-6-phosphate isomerase (GPI)
D-fructose 6-phosphate (Fru-6-P)	C6H13O9P	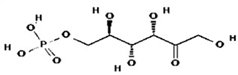	GPI	Phosphomannose isomerase (MPI)
D-mannose 6-phosphate (Man-6-P)	C6H13O9P	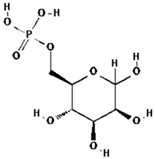	MPI	PMM2
D-mannose 1-phosphate (Man-1-P)	C6H13O9P	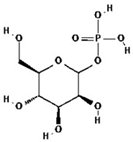	PMM2	GDP-MP (GMPPA/GMPPB complex)
GDP-D-mannose (GDP-Man)	C16H25N5O16P2	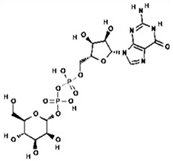	GDP-MP	GMDS
GDP-4-keto-6-deoxy-D-mannose	C16H23N5O15P2	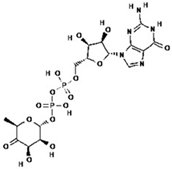	GMDS	TSTA3 (FX)
GDP-L-fucose (GDP-L-Fuc)	C16H25N5O15P2	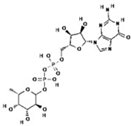	TSTA3/GFPP	FUTs / POFUTs
L-fucose (L-Fuc)	C6H12O5	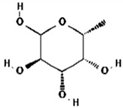	—	Fucokinase (FUK)
L-fucose 1-phosphate (Fuc-1-P)	C6H13O8P	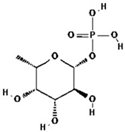	FUP	GFPP

## Data Availability

No new data were created or analyzed in this study.

## Abbreviations

Abbreviation	Full name
AGP	Alpha-1-acid glycoprotein
AHR	Aryl hydrocarbon receptor
CAC	Colitis-associated cancer
CDC	Complement-dependent cytotoxicity
CRC	Colorectal cancer
CRP	C-reactive protein
CSCs	Colon cancer stem cells
CT	Cholera toxin
CTLs	Cytotoxic T lymphocytes
DSS	Dextran sodium sulfate
ECM	Extracellular matrix
EGFR	Epidermal growth factor receptor
EMT	Epithelial-mesenchymal transition
FA	Food allergy
FKP	Fucokinase/GDP-L-fucose pyrophosphorylase
GDP-MP	GDP-mannose pyrophosphorylase
FUCA	α-L-Fucosidase
GOLPH3	Golgi phosphoprotein 3
GSL	Glycosphingolipid
FUTs	Fucosyltransferases
GMDS	GDP-D-mannose 4, 6-dehydratase
HSF1	Heat shock factor 1
HFD	High-fat diet
HMOs	Human milk oligosaccharides
IBD	Inflammatory bowel disease
IECs	Intestinal epithelial cells
ISCs	Intestinal stem cells
LPS	Lipopolysaccharide
NEC	Necrotizing enterocolitis
PMM2	Phosphomannomutase 2
SCFAs	Short-chain fatty acids
SLE	Systemic lupus erythematosus
Tregs	Regulatory T cells
TLR	Toll-like receptor
